# High-Fidelity and Cost-Effective Engineering of SARS-CoV-2

**DOI:** 10.3390/v17121604

**Published:** 2025-12-11

**Authors:** Marco Olguin-Nava, Thomas Hennig, Charlene Börtlein, Patrick Bohn, Uddhav B. Ambi, Alexander Gabel, Lina M. Günter, Anne-Sophie Gribling-Burrer, Nora Schmidt, Neva Caliskan, Lars Dölken, Mathias Munschauer, Redmond P. Smyth

**Affiliations:** 1 Helmholtz Institute for RNA-Based Infection Research, Helmholtz Centre for Infection Research, 97080 Würzburg, Germany; olguinnava.marco@mh-hannover.de (M.O.-N.);; 2 Institute of Virology, Hannover Medical School, 30625 Hannover, Germany; 3 Institute of Virology and Immunology, University of Würzburg, 97080 Würzburg, Germany; 4 Institute of Medical Virology, Goethe-University Frankfurt, 60596 Frankfurt am Main, Germany; 5 Architecture et Réactivité de l′ARN, Institute of Molecular and Cellular Biology, CNRS, University of Strasbourg, 67084 Strasbourg, France; 6 Hannover Medical School, 30625 Hannover, Germany; 7 Faculty of Medicine, University of Würzburg, 97080 Würzburg, Germany; 8 Faculty of Biology and Preclinical Medicine, University of Regensburg, 93053 Regensburg, Germany; 9 Heidelberg University, Medical Faculty Heidelberg, Department of Infectious Diseases, Molecular Virology, Center for Integrative Infectious Disease Research, 69120 Heidelberg, Germany

**Keywords:** SARS-CoV-2, reverse genetic system, spike, NSP9

## Abstract

Efficient reverse genetics systems are essential for understanding SARS-CoV-2 pathogenesis, host–virus interactions, and potential therapeutic interventions. Here, we developed a cost-effective PCR-based reverse genetics platform that splits the SARS-CoV-2 genome into only six bacterial plasmids, enabling cloning, manipulation, and the rescue of recombinant SARS-CoV-2 (rSARS-CoV-2) with high fidelity and high viral titers after a single passage. Using this system, we generated and characterized spike protein mutants Y453F and N501Y, as well as a U76G mutation in the 5′-UTR. Y453F showed reduced replication kinetics, lower cell binding, and diminished fitness, while N501Y exhibited comparable replication and fitness, highlighting the distinct effects of these spike protein mutations. The U76G mutation is located within a novel NSP9 binding site in the 5′-UTR and leads to impaired RNA synthesis and reduced viral replication efficiency, suggesting an important role in transcription and replication. Our findings highlight the robustness and adaptability of this reverse genetics system, providing a versatile, cost-effective tool for studying SARS-CoV-2 mutations and their effects on replication and fitness, with potential applications in vaccine and therapeutic development.

## 1. Introduction

Severe acute respiratory syndrome coronavirus 2 (SARS-CoV-2) is the causative agent of the respiratory illness COVID-19, which emerged in December 2019 and has since unleashed a pandemic that continues to impact human health [1]. SARS-CoV-2 possess a large (30 kb), capped and polyadenylated positive sense genome comprised of numerous open reading frames (ORFs) and flanked by two terminal untranslated regions (UTRs). The 5′ and 3′ UTRs are around 250 to 360 bp and highly structured [2,3]. The 5′-terminal region contains two overlapping ORFs, ORF1a and ORF1b, which encode for two polyproteins, pp1a and pp1ab, that are cleaved into 16 non-structural proteins (NSPs) with multiple activities required for viral infection [2,4]. Pp1ab is translated using a minus 1 programmed ribosomal frameshift event to produce the RNA-dependent RNA polymerase (RdRp). Meanwhile, the 3′-terminal region (one-third of the genome) encodes the structural proteins which are expressed from subgenomic mRNAs (sgmRNAs) [5]. Like many RNA viruses, SARS-CoV-2 exhibits rapid rates of evolution [6,7,8,9]. Mutations arising in the SARS-CoV-2 genome, especially in “variants of concern”, have been shown to impact infectivity, transmissibility or evasion of the immune system compared to the original strain [7,10,11]. Consequently, despite the availability of highly effective vaccines [12,13,14], high infection rates persist worldwide.

A reverse genetics system (RGS) is a key tool required for molecular virology as it enables the generation of recombinant viruses to study various aspects of viral biology, including interactions with host cells [15,16,17]. RGS can be employed to investigate mutant virus behavior, screen antiviral drugs, develop therapeutic strategies, facilitate diagnostics [18,19,20,21,22,23,24], and generate attenuated viruses for vaccine development [15,25,26,27]. Despite their utility for virus research, RGS have been challenging to establish for viruses with large genomes, such as SARS-CoV-2. Cloning long viral sequences into bacterial plasmids is hindered by issues of instability and toxicity [16]. Furthermore, in vitro transcription of long RNA transcripts and their efficient transfection into mammalian cells pose a significant technical challenge [16].

Bacterial Artificial Chromosome (BAC) [23,28] and Yeast Artificial Chromosome (YAC) [29,30] are high-capacity vectors capable of propagating very large viral sequences in bacteria and yeast, respectively. Transfection or electroporation of BAC and YAC has been used for the rescue of SARS-CoV-2 infectious molecular clones [18,19,23,31]. However, BAC and YAC are challenging to construct, and their subsequent genetic manipulation is more complex compared to that of high- or low-copy small bacterial plasmids. Alternatively, the SARS-CoV-2 genome can be partitioned into fragments, cloned into bacterial plasmids, and reassembled either in vitro or in cells [22,32,33,34,35,36,37]. While this approach facilitates genetic manipulation using widely established techniques, it requires precise reassembly of the DNA fragments to form a contiguous genome. High-fidelity reassembly of SARS-CoV-2 genome has been achieved using Type IIS restriction enzymes, which cleave outside their recognition sequences for scarless and directional assembly, in a strategy known as Golden Gate-like assembly [22,32,33,34]. Alternatively, circular polymerase extension reaction (CPER) generates overlapping SARS-CoV-2 genome fragments, which are subsequently assembled into the complete genome in a PCR-like reaction [35,36,38].

**Figure 1:**
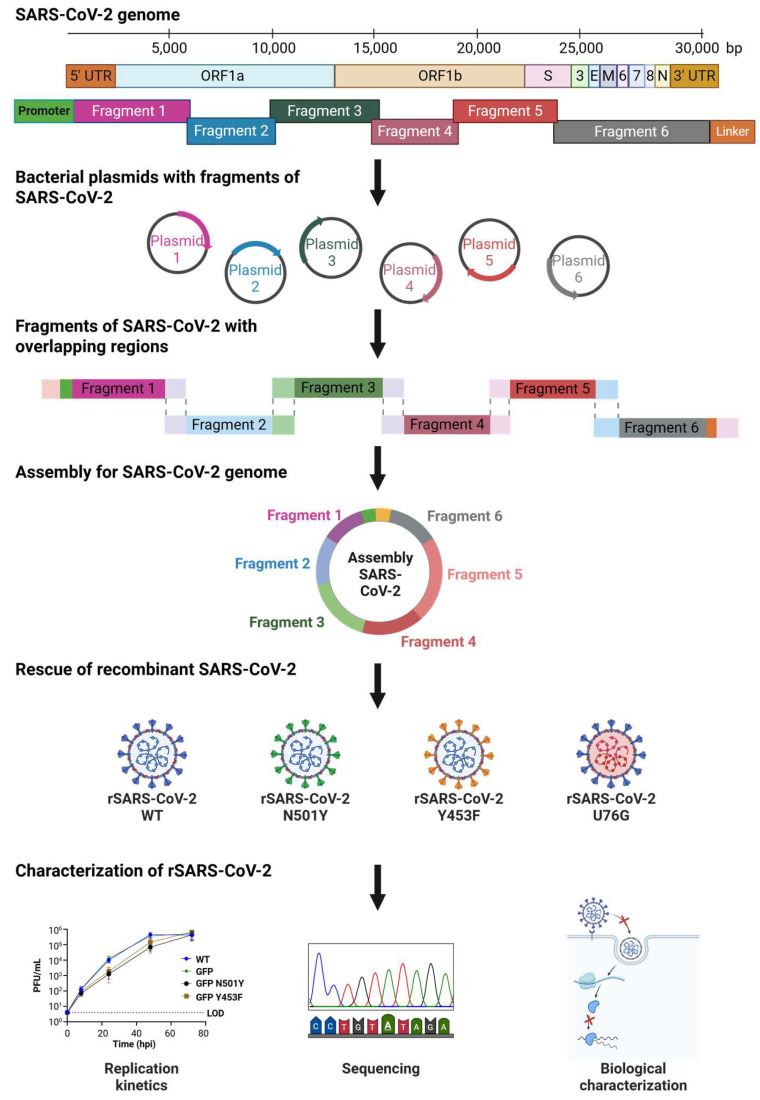
General overview of PCR-Based Universal Reverse Genetics System for SARS-CoV-2. The SARS-CoV-2 genome is divided into six fragments (F1 1–5682, F2 5682–8869, F3 8864–14,487, F4 14,483–17,992, F5 17,948–24,119, F6 24,089–29,891) and cloned into bacterial plasmids. Each fragment is amplified with 20 bp overlapping ends to facilitate the assembly of the complete viral genome by PCR. Following assembly, the SARS-CoV-2 DNA is transfected into mammalian cells to rescue the recombinant virus for molecular characterization.

Here, we established a SARS-CoV-2 reverse genetics system based on the splitting and cloning of the viral genome into six individual fragments, which were subsequently reassembled by a PCR-like reaction (Figure 1). We divided and cloned the full SARS-CoV-2 genome into six plasmids, one of the smallest fragment numbers reported to date, enabling stable propagation and genetic manipulation using well-established cloning methodologies [39]. The PCR assembled SARS-CoV-2 genome was then transfected into mammalian cells using polyethylenimine (PEI), a low-cost and easily accessible reagent. We applied this strategy to generate two rSARS-CoV-2 spike mutant viruses, Y453F and N501Y, which were first identified in variants of concern [7]. In addition, we generated an rSARS-CoV-2 containing the non-coding U76G mutation in the 5′UTR of the SARS-CoV-2 genome. The recombinant viruses were characterized, revealing distinct phenotypic effects when tested for replication, cell binding, competition assays and subgenomic mRNA synthesis.

## 2. Materials and Methods

### 2.1. Cells

All cell lines were cultured at 37 °C in a humidified atmosphere containing 5% CO_2_ in Dulbecco′s Modified Eagle Medium (Gibco, Thermo Fisher Scientific, Waltham, MA, USA) supplemented with 10% fetal bovine serum (Sigma-Aldrich, St. Louis, MO, USA) and 100 U/mL penicillin–streptomycin (Gibco, Thermo Fisher Scientific, Waltham, MA, USA). Vero E6 TMPRSS2 cells (African green monkey kidney; a generous gift from S. Pöhlmann), A549 ACE2 cells (human lung carcinoma epithelial cells; a generous gift from A. Pichlmair), and HEK293 cells (human embryonic kidney; a generous gift from U. Fischer) were used in this study. HEK293 ACE2 cells were generated by retroviral transduction based on the plasmid pWPI-ACE2-puro (a generous gift from A. Pichlmair), followed by puromycin selection. All cell lines were routinely tested and confirmed to be free of mycoplasma contamination. 

### 2.2. Plasmids

The full genome of SARS-CoV-2 WT was split into 6 fragments (from 6.2 to 3.2 kb) which were amplified using as a template the YAC pCC1BAC-HIS3-SARS-CoV-2 (GenBank MN996528.1) and for SARS-CoV-2 GFP an extra fragment using as a template the YAC pCC1BAC-HIS3-SARS-CoV-2-GFP (BioProject: PRJNA615319; BioSample: SAMN14450690; Sample name: GFP-2_rSARS-CoV-2), both clones were kindly provided by Prof. Dr. Volker Thiel. The amplification was performed using specific primers (Supplementary Table S1) and high-fidelity PrimeSTAR GXL DNA polymerase (Takara Bio Inc. Kusatsu, Shiga, Japan). After the generation of 6 fragments, fragment 4 (3.5 kb) was cloned into the high copy number plasmid (PUC19) using restriction enzymes BamHI and KpnI (New England Biolabs, Ipswich, MA, USA). Meanwhile the remaining six fragments (6.2 to 3.2 kb) were cloned into pUA66 to improve stability. The following restriction enzymes were used during the cloning: BamHI and KpnI (New England Biolabs, Ipswich, MA, USA) for fragment 1 and fragment 2, BamHI HF and SacI (NEB) for fragment 3, XbaI and BamHI (New England Biolabs, Ipswich, MA, USA) for fragment 5 and XmaI (New England Biolabs, Ipswich, MA, USA) for fragment 6.

The following plasmids were then modified: for plasmid F1 the initial promoter T7 was removed and replaced by a CMV promoter. In the case of fragment F6 WT and F6 GFP a linker sequence was added to the 3′ UTR using restriction enzymes KpnI and Esp3I (New England Biolabs, Ipswich, MA, USA). The linker was de novo synthesized (IDT) and contains the next elements: (1) Hepatitis delta virus ribozyme (HDVr), (2) SV40 poly (A) signal and (3) a spacer sequence of 364 bp.

Mutations Y453F and N501Y were inserted in plasmid F5 by Directed Site Mutagenesis using specific primers (Supplementary Table S1) and high-fidelity PrimeSTAR GXL DNA polymerase (Takara Bio Inc. Kusatsu, Shiga, Japan). Similar strategy was followed for the mutation T76G in plasmid F1.

An individual plasmid containing the nucleoprotein (N) of SARS-CoV-2 was generated using the clone pCC1BAC-HIS3-SARS-CoV-2 as a template. The N sequence of 1260 bp was amplified using specific primers (Supplementary Table S1) and cloned downstream a CMV promoter into the vector PUC19.

Plasmids were propagated in NEB stable competent *E. coli* (New England Biolabs, Ipswich, MA, USA) grown overnight at 30 °C. All plasmids were subjected to Sanger sequencing using a set of primers (Supplementary Table S1), only a single silent point mutation C28103A located in plasmid 6 WT was detected compared to the sequence of SARS-CoV-2 isolate Wuhan-Hu-1 (GenBank MN996528.1).

### 2.3. PCR Amplification of SARS-CoV-2 Individual Fragments

Each individual SARS-CoV-2 fragment was amplified from their respective plasmids using an exclusive pair of primers (Supplementary Table S1) and high-fidelity PrimeSTAR GXL DNA polymerase (Takara Bio Inc. Kusatsu, Shiga, Japan), followed by gel isolation with NucleoSpin Gel and PCR Clean-up (Macherey-Nagel, Düren, North Rhine-Westphalia, Germany) following the manufacturer′s recommendations. PCR conditions consisted in 0.05 U of PrimeSTAR GXL polymerase (Takara Bio Inc. Kusatsu, Shiga, Japan), 250 nM of each primer, 200 µM of each dNTP and 1 × PrimerSTAR GXL buffer in a total volume of 50 µL. Cycling conditions were initial denaturation for 2 min at 98 °C, followed by 35 cycles for 10 sec at 98 °C, 15 sec at 55 °C, and 10 min at 68 °C, followed by a final extension for 15 min at 68 °C. Amplicon quality was checked on 1% agarose gel post-stained in EtBr.

### 2.4. SARS-CoV-2 Genome Assembly by Polymerase Extension

SARS-CoV-2 genome was assembled using the following PCR-based protocol (Takara Bio Inc. Kusatsu, Shiga, Japan). Each fragment possesses complementary ends of 20 nucleotides overlap used for the assembly. The six fragments were mixed in equimolar amount of 0.1 pM, 2 µL of PrimeSTAR GXL DNA polymerase (Takara Bio Inc. Kusatsu, Shiga, Japan), 200 µM of each dNTP, 1x GXL buffer into a final volume of 50 µL.

Two initial cycling conditions for SARS-CoV-2 WT and SARS-CoV-2 GFP were tested: (1) initial denaturation at 98 °C for 30 s, 12 cycles of 10 s at 98 °C, 20 s at 55 °C and 10 min at 68 °C, and a final elongation for 12 min at 68 °C. (2) initial denaturation at 98 °C for 2 min, 20 cycles of 10 s at 98 °C, 15 s at 55 °C and 25 min at 68 °C, and a final elongation for 25 min at 68 °C. Assembly quality was checked on 0.5% agarose gel post-stained in EtBr.

We performed an optimization of the number of cycles using 6, 9 or 12 cycles. The condition using 12 cycles performed the best results. The same conditions were used to generate all rSARS-CoV-2 viruses, exchanging the corresponding fragment with the fragment containing the desired mutation. Assembly reactions were then used for electroporation, nucleofection or transfection without any purification step.

### 2.5. DNA Electroporation

Unpurified SARS-CoV-2 GFP DNA assembly and N plasmid were electroporated into HEK293T and HEK293T ACE2 cells. Briefly, 1 × 10^6^ HEK293T or HEK293T ACE2 cells were resuspended in cold Opti-MEM (Gibco, Thermo Fisher Scientific, Waltham, MA, USA) completed with ATP (Thermo Fisher Scientific, Waltham, MA, USA) and glutathione (Sigma-Aldrich, St. Louis, MO, USA). Then, the cell suspension was mixed with 50 µL of SARS-CoV-2 GFP DNA assembly and 200 ng of N plasmid. The mixture was transferred into a 4 mm electroporation cuvette (Bio-Rad Laboratories, Hercules, CA, USA) and placed into the Gene Pulser Xcell electroporation system (Bio-Rad Laboratories, Hercules, CA, USA). A single electrical pulse with settings of 270 V at 950 μF was performed. Next, the mixture was incubated for 5 min at RT and later transferred into a Falcon tube containing 1.5 mL Dulbecco′s Modified Eagle Medium DMEM (Gibco, Thermo Fisher Scientific, Waltham, MA, USA) with 10% fetal bovine serum (Sigma-Aldrich, St. Louis, MO, USA) and 100 U/mL of penicillin-streptomycin (Gibco, Thermo Fisher Scientific, Waltham, MA, USA). Cells were incubated under 5% CO_2_ and 37 °C conditions. After 24 h, cells were trypsinized and seeded over a monolayer of 5 × 10^5^ Vero E6 TMPRSS2 cells. Cells were incubated under 5% CO_2_ and 37 °C conditions and observation for GFP expression was performed every 24 h for 10 days.

### 2.6. DNA Nucleofection

Unpurified SARS-CoV-2 GFP DNA assembly and N plasmid were nucleofected into HEK293T or HEK293T ACE2 cells using program CM-130 in the 4D-Nucleofector System (Lonza, Basel, Switzerland). Then, 2 × 10^5^ HEK293T or HEK293T ACE2 cells were resuspended in 20 µL of 4D-Nucleofector Solution (Lonza, Basel, Switzerland), mixed with 50 µL of SARS-CoV-2 GFP DNA assembly and 200 ng of N plasmid. The mixture was placed in Nucleocuvette Vessel (Lonza, Basel, Switzerland) and processed using program CM-130. After the end of program, cells were resuspended in 100 µL warm Dulbecco′s Modified Eagle Medium DMEM (Gibco, Thermo Fisher Scientific, Waltham, MA, USA) with 10% fetal bovine serum (Sigma-Aldrich, St. Louis, MO, USA) and 100 U/mL of Penicillin-Streptomycin (Gibco, Thermo Fisher Scientific, Waltham, MA, USA) and then were incubated under 5% CO_2_ and 37 °C conditions. After 24 h, cells were trypsinized and seeded over a monolayer of 5 × 10^5^ Vero E6 TMPRSS2 cells. Cells were incubated under 5% CO_2_ and 37 °C conditions and observation for GFP expression was performed every 24 h for 10 days.

### 2.7. DNA Transfection Using Commercial Transfection Reagents

Reverse transfection of SARS-CoV-2 DNA assembly and N plasmid into HEK293T and HEK293T ACE2 cells was performed using different commercial reagents. Briefly, 50 µL of SARS-CoV-2 GFP DNA assembly and 200 ng of N plasmid were mixed with Lipofectamine 2000 (Thermo Fisher Scientific), Lipofectamine 3000 (Thermo Fisher Scientific), TransIT 2020 (Mirus Bio, Madison, WI, USA) and TransIT LT1 (Mirus Bio, Madison, WI, USA following the manufacturer′s recommendations. The mixture was added in a 6 well plate and 1 × 10^6^ HEK293T or HEK293T ACE2 cells were dropped on the top of the DNA: transfection reagent mixture. Cells were incubated under 5% CO_2_ and 37 °C conditions. After 24 h, cells were trypsinized and seeded over a monolayer of 5 × 10^5^ Vero E6 TMPRSS2 cells. Cells were incubated under 5% CO_2_ and 37 °C conditions and observation for GFP expression was performed every 24 h for 10 days.

### 2.8. Virus Rescue by DNA Transfection Using PEI

Virus rescue was accomplished by reverse transfection of SARS-CoV-2 DNA assembly and N plasmid into HEK293T ACE2 cells. As an initial test different conditions were tested. Briefly, 25 µL of SARS-CoV-2 GFP DNA assembly without purification and 200 ng of N plasmid were mixed with 7.2 or 14.4 µL of polyethylenimine (PEI) (Polysciences, Warrington, PA, USA) or 50 µL of SARS-CoV-2 GFP DNA without purification and 200 ng of N plasmid were mixed with 14.4 or 28.8 µL of PEI. Then, the mixture was placed in a 6 well plate and after 10 min of incubation at RT, 1 × 10^6^ HEK293T or HEK293T ACE2 cells were dropped on the top of the DNA: PEI mixture. Cells were incubated under 5% CO_2_ and 37 °C conditions. After 24 h, cells were trypsinized and seeded over a monolayer of 5 × 10^5^ Vero E6 TMPRSS2 cells. Cells were incubated under 5% CO_2_ and 37 °C conditions and observation for GFP expression was performed every 24 h for 10 days.

For subsequent rescues of rSARS-CoV-2 WT and mutant viruses, the only condition used was the reverse transfection of 50 µL of SARS-CoV-2 DNA assembly mixed with 200 ng of N plasmid and 28.8 µL of PEI into HEK293T ACE2 cells. Recombinant viruses were amplified once on Vero E6 TMPRSS2 to generate viral stocks.

### 2.9. Production of Stocks for rSARS-CoV-2

First, 8 × 10^6^ Vero E6 TMPRSS2 cells were infected using rSARS-CoV-2 at a MOI 0.1 for 1 h at 37 °C. After, the inoculum was removed and 15 mL of DMEM supplemented with 5% FCS, 100 U/mL of penicillin–streptomycin was added. Then, 48 hpi supernatant was removed and centrifugated at 3000 × *g* for 10 min. Without disturbing the cell pellet, supernatant was taken and 500 µL aliquots were generated. Each aliquot was immediately storage at −70 °C.

### 2.10. Viral Infections

In general, rSARS-CoV-2 inoculums were prepared in DMEM supplemented with 1% FCS. Before the infection, Vero E6 TMPRSS2 or A549 ACE2 cells were washed once with PBS and incubated with the respective inoculum for 1 h at 37 °C with gentle shaking every 10 min. The inoculum was removed and fresh DMEM supplemented with 5% FCS, 100 U/mL of penicillin–streptomycin was added to the cells.

### 2.11. Replication Kinetics for rSARS-CoV-2

Replication kinetics for the different rSARS-CoV-2 were performed using Vero E6 TMPRSS2 or A549 ACE2 cells with a MOI 0.01 PFU/cell. Samples collected at 8, 24, 28 and 72 hpi were titrated in duplicate by plaque assay. Statistical analysis comparison was performed using two-sided unpaired Student′s *t*-test with Prism 7 (GraphPad Software Inc, San Diego, CA, USA).

### 2.12. Plaque Assay

Cell supernatant containing virus was 10-fold serial diluted in DMEM 1% FCS, inoculated onto TMPRSS2-Vero E6 cell monolayer in duplicate, incubated at 37 °C for 1 h. After the incubation, the inoculum was removed and the cell monolayer was overlayed with 0.6% (*w*/*v*) methylcellulose (Carl Roth GmbH, Karlsruhe, Germany) in MEM (Gibco, Thermo Fisher Scientific, Waltham, MA, USA) supplemented with 25 mM of HEPES, 0.44% NaHCO_3_, 2 mM of GlutaMAX (Gibco, Thermo Fisher Scientific, Waltham, MA, USA), 100 U/mL of penicillin–streptomycin and 5% FCS and incubated at 37 °C. After 3 days, cells were fixed and stained with 2x staining solution (0.23% crystal violet, 8% formaldehyde, 10% ethanol) directly to the medium for 24 h. Cells were washed twice with H_2_O and plaques enumerated to determine viral titers.

### 2.13. RNA Extraction, cDNA Synthesis and qPCR

Total RNA was extracted with Trizol (Invitrogen, Carlsbad, CA, USA) using the manufacturer′s recommendations and the totality of the viral RNA was treated with Turbo DNase (Thermo Fisher Scientific, Waltham, MA, USA) for 30 min at 37 °C. Following DNase treatment, RNA was column purified using NTC buffer and the NucleoSpin Gel and PCR Clean-up kit (Macherey-Nagel, Düren, North Rhine-Westphalia, Germany), according to the manufacturer′s instructions.

The RNA was then reverse transcribed using SSIV (Thermo Fisher Scientific, Waltham, MA, USA) with a set of random hexamers (Integrated DNA Technologies, Coralville, IA, USA). Quantitative real-time PCR (qRT-PCR) was performed using PowerUP SYBR green (Thermo Fisher Scientific, Waltham, MA, USA) according to manufacturer′s instructions. A standard curve for *N* and *RdRp* was made using serial dilutions of plasmids F6 and F4, respectively. The oligonucleotides used to amplify *N*, *RdRp*, *TMPRSS2*, *ACE2* and *18S rRNA* are described in Supplementary Table S1. The level of each RNA was determined by CFX96 Touch Real-Time PCR Detection System (Bio-Rad) with the cycling condition: 50 °C for 2 min, 95 °C for 2 min, followed by 40 cycles of 95 °C for 15 s and 60 °C for 30 s, finishing with melt profile analysis. The software used for data statistical analysis is Prism 7 (GraphPad Software Inc, San Diego, CA, USA).

### 2.14. cDNA Synthesis and Sanger Sequencing for Spike Mutants and 5′ UTR Mutant

After RNA extraction and DNase treatment, the RNA was reverse transcribed for 4 h using SSIV (Thermo Fisher Scientific, Waltham, MA, USA) with a set of reverse SARS-CoV-2 primers (Supplementary Table S1). An amplicon of 2.4 kb was produced using the previous cDNA and a specific pair of primers (Supplementary Table S1) for the T76G, Y453F and N501Y using the following conditions: 1 µL of diluted 1/10 RT reaction with 0.05 µL of PrimeSTAR GXL polymerase (Takara Bio Inc. Kusatsu, Shiga, Japan), 250 nM of each primer, 200 µM of each dNTP and 1 x PrimerSTAR GXL buffer in a total volume of 25 µL. Cycling conditions were initial denaturation for 1.5 min at 98 °C, followed by 35 cycles for 10 s at 98 °C, 15 s at 55 °C, and 3 min at 68 °C, with a final extension for 5 min at 68 °C. Amplicon quality was checked on 1% agarose gel post-stained in EtBr.

PCR products were purified using NucleoSpin Gel and PCR Clean-up (Macherey-Nagel, Düren, North Rhine-Westphalia, Germany) according to manufacturer′s recommendations. Spike mutant PCR products for Y453F and N501Y were analyzed by Sanger sequencing using the specific primer (TTCAGCCCCTATTAAACAGCCTGCACGTGT), meanwhile PCR product for T76G was analyzed by Sanger sequencing with the specific primer (GGCAAAACGCCTTTTTCAACTTC).

### 2.15. Nanopore Sequencing and Bioinformatics Analysis

Supernatant of infected Vero E6 TMPRRS2 cells were treated for RNA extraction using Trizol (Invitrogen, Carlsbad, CA, USA) based on the manufacturer′s recommendations, followed by Ethanol/Sodium Acetate precipitation and resuspension in RNase-free H_2_O. Then, 10 µg of RNA were treated with Turbo DNase (Thermo Fisher Scientific, Waltham, MA, USA) for 30 min at 37 °C. Following DNase treatment, RNA was column purified using the NucleoSpin Gel and PCR Clean-up kit (Macherey-Nagel, Düren, North Rhine-Westphalia, Germany) with NTC buffer according to the manufacturer′s instructions.

Reverse transcription was performed using in-house purified MarathonRT. pET-6xHis-SUMO-MarathonRT encoding MarathonRT was a gift from Anna Pyle (Addgene plasmid # 109029; http://n2t.net/addgene:109029 (accessed on 1 May 2025); RRID: Addgene_109029). Viral RNA was reverse transcribed using a mix of reverse primers listed in Table S1. Specifically, RNA was mixed with 0.5 mM dNTPs, 5 µM of each primer in 9 µL total volume and denatured for 5 min at 65 °C. Samples were placed on ice for 2 min and reverse transcription was initiated by adding 40 U of MarathonRT in 50 mM Tris-HCl pH 8.3, 200 mM KCl, 20% glycerol (*v*/*v*), 1 mM MnCl_2_, 4 U of RNasin in a 20 µL total volume. Samples were incubated for 4 h at 42 °C. Controls lacking reverse transcriptase were carried out as above, with the omission of the MarathonRT enzyme.

The RT product was subsequently used in a set of 14 separate PCR reactions to produce 2.4 kb amplicons that covered the full genome of SARS-CoV-2. PCR amplification conditions were 1 µL of diluted 1/10 RT reaction with 0.05 U of PrimeSTAR GXL polymerase (Takara Bio Inc. Kusatsu, Shiga, Japan), 250 nM of each primer, 200 µM of each dNTP and x1 PrimerSTAR GXL buffer in a total volume of 25 µL, using the cycling conditions in Section 2.14. Amplicon quality was checked on 1% agarose gel post-stained in EtBr.

PCR products were pooled and purified using Mag-Bind Totalpure NGS beads (Omega Bio-tek, Norcross, GA, USA) by addition of 0.6 × volumes of beads followed by light agitation for 5 min at room temperature. Beads were pelleted on a magnetic rack (Invitrogen, Carlsbad, CA, USA), followed by removal of supernatant and 2 washes with 100 µL freshly prepared 70% ethanol. Finally, beads were air dried for 3–5 min (until appearance changed from glossy to rough) and DNA was eluted by addition of 30 µL H_2_O, followed by 5 min incubation at room temperature.

DNA concentration was quantified via Nanodrop, and 300 ng of pooled product was taken into the Nanopore native ligation barcoding library preparation. Specifically, the DNA in 11.5 µL in a PCR tube was end-repaired by addition of 1.75 µL NEB Ultra II End Repair Buffer (New England Biolabs, Ipswich, MA, USA) and 0.75 µL Enzyme Mix followed by thorough mixing via pipetting, incubated for 5 min at room temperature and 5 min at 65 °C. Next, 1 µL of end-repaired DNA was transferred into a new PCR tube, followed by addition of 0.75 µL H_2_O, 1.25 µL native ligation barcode (ONT SQK-NBD114-96) and 3 µL Blunt/TA Ligase Master Mix (New England Biolabs, Ipswich, MA, USA). The reaction was mixed by pipetting, incubated for 20 min at room temperature, and terminated by addition of 1 µL EDTA (SQK-NBD114-96). The barcoded DNA samples were then pooled and purified by addition of 0.4 volumes of SPRI beads (SQK-NBD114-96). For binding, they were incubated for 5 min with mixing, then pelleted on a magnetic rack. Next, beads were washed twice by resuspension and re-pelleting in 200 µL Short Fragment Buffer (SFB, ONT SQK-NBD114-96) before a final wash step with 100 µL 80% ethanol. After a brief drying on air, the DNA was then eluted by addition of 35 µL and incubation for 10 min at 37 °C with mixing. Finally, 30 µL of the barcoded DNA was ligated onto 2.5 µL Native Ligation Adapter (NA, ONT SQK-NBD114-96) by addition of 12.5 µL NEBNext Quick Ligation Reaction Buffer (New England Biolabs, Ipswich, MA, USA) and 5 µL high conc. T4 DNA Ligase (New England Biolabs, Ipswich, MA, USA). After incubation for 20 min at room temperature, 20 µL of Ampure XP Beads (SQK-NBD114-96) were added, followed by incubation for 10 min at room temperature with mixing, pelleting of beads in a magnetic rack, and two washes with 125 µL SFB (SQK-NBD114-96). After removal of wash buffer, the beads were resuspended in 7 µL Elution Buffer (SQK-NBD114-96), incubated for 10 min at 37 °C, followed by pelleting and transfer of the supernatant into a 1.5 mL DNA LoBind tube. The prepared library was quantified with AccuClear Ultra High Sensitivity dsDNA kit (Biotium, Fremont, CA, USA) and 14 ng (approximately 10 fmol) were loaded onto a Kit 14 Flongle flow cell (FLO-FLG114). Sequencing data were acquired with MinKNOW version 22.12.7 (4 kHz sampling) for the rescue verification and 23.07.8 (5 kHz sampling) for the competition assay, respectively.

All data were subsequently basecalled and demultiplexed with dorado v0.5.0 (min-qscore 11) and aligned to the respective SARS-CoV-2 wildtype (NC045512.2) or SARS-CoV-2 GFP (BioProject: PRJNA615319; BioSample: SAMN14450690) reference sequence with LAST v1450. The generated maf files were then converted into sorted and indexed bam files using samtools v1.16.1, and mutation frequencies for each position with a coverage of at least 100 were quantified with perbase v0.8.5. The generated tables were then parsed into pandas (v1.4.3) dataframes. Figures were generated with the python package plotly v5.9.0.

### 2.16. Western Blot

For Western blot analysis, 5 × 10^5^ cells were rinsed with PBS and lysed in 100 μL Laemmli buffer (2% SDS, 10% Glycerol, 60 mM Tris, 0.01% (*w*/*v*) bromphenol blue, 50 mM DTT) and lysates were sheared by passing through a syringe. Proteins were separated by SDS PAGE in NuPAGE 4–12% Bis-Tris Protein Gels (Thermo Fisher Scientific, Waltham, MA, USA) and transferred to a nitrocellulose membrane using the iBlot dry blotting system (Thermo Fisher Scientific, Waltham, MA, USA). Membranes were incubated with anti-N (cat. GTX135357, Cell Signaling Technology, Danvers, MA, USA), anti-ACE2 (cat. 66699, Proteintech Group, Rosemont, IL, USA), anti-TMPRSS2 (cat. PA5-14264, Thermo Fisher Scientific, Waltham, MA, USA), or anti-Actin (cat. sc-47778, Santa Cruz Biotechnology, Dallas, TX, USA) antibodies. We used the following secondary antibodies: IRDye 800CW goat anti-rabbit IgG (LI-COR Biosciences, Lincoln, NE, USA), IRDye 680RD goat anti-rabbit IgG (LI-COR), IRDye 800CW donkey anti-goat IgG (LI-COR Biosciences) and IRDye 800CW goat anti-mouse IgG (LI-COR Biosciences) using the iBind Automated Western System (Thermo Fisher Scientific, Waltham, MA, USA). The membranes were imaged in the Odyssey Clx Infrared Imager System (LI-COR Biosciences). Statistical analysis comparison between the blots were performed using one-way ANOVA with Prism 7 (GraphPad Software Inc, San Diego, CA, USA).

### 2.17. Binding Assay

First, 5 × 10^5^ Vero E6 TMPRSS2 and A549 ACE2 cells were washed twice with cold PBS and then incubated with rSARS-CoV-2 WT, rSARS-CoV-2 Y453F or rSARS-CoV-2 N501Y using at MOI 0.1 or 0.01 PFU/cell for1 h at 4 °C with gentle shaking every 10 min. Then, the inoculum was removed, and cells were washed twice with cold PBS and cells were lysed with TRIZOL (Invitrogen, Carlsbad, CA, USA). RNA extraction, RT and qPCR for *18S rRNA* and *N* were performed as described before. To calculate differences in RNA expression we used the DDCT method versus *18S rRNA*. Statistical analysis comparison between the bindings of each RDP spike mutant against rSARS-CoV-2 WT was performed using one-way ANOVA with Prism 7 (GraphPad Software Inc, San Diego, CA, USA).

### 2.18. Competition Assay

Competition assay was performed infecting a monolayer of 5 × 10^5^ Vero E6 TMPRSS2 or A549 ACE2 cells with 5 different virus combinations at a MOI 0.1 PFU/cell: (1) rSARS-CoV-2 WT, (2) rSARS-CoV-2 Y453F, (3) rSARS-CoV-2 N501Y, (4) rSARS-CoV-2 WT plus rSARS-CoV-2 Y453F or 5) rSARS-CoV-2 WT plus rSARS-CoV-2 N501Y for 1 h at 4 °C with gently shake every 10 min. After, the inoculum was removed, fresh DMEM supplemented with 5% FCS, 100 U/mL of penicillin–streptomycin was added to the cells. Cells were incubated under 5% CO_2_ and 37 °C conditions.

After 2 days, cell monolayers were lysed using TRIZOL. RNA extraction and RT using Marathon RT were performed as described before. Next, each sample was PCR amplified to produce a 2.4 kb amplicon using primers (Fw primer: ACAAATCCAATTCAGTTGTCTTCCTATTC and Rv primer: TGTGTACAAAAACTGCCATATTGCA). Library preparation, nanopore sequencing and data analysis were carried out as described in the section for nanopore sequencing and bioinformatics analysis. For both cell lines Vero E6 TMPRSS2 and A549 ACE2, two independent experiments with technical replicates were performed.

### 2.19. gRNA and sgRNA Quantification

First, 5 × 10^5^ Vero E6 TMPRSS2 cells were infected with rSARS-CoV-2 WT or rSARS-CoV-2 at a MOI 0.01 PFU/cell collecting samples at 8, 24, 48 and 72 hpi. After RNA extraction and DNase treatment described as before, RNA was reverse transcribed using SSIV (Thermo Fisher Scientific, Waltham, MA, USA) with a set of random hexamers (Integrated DNA Technologies). To specifically analyze viral sgRNAs and gRNA, we used qRT-PCR with previously designed primers [40]: a forward primer that binds within the SARS-CoV-2 leader sequence and a specific reverse primer for the ORF1a RNA, the M mRNA or the N mRNA (Supplementary Table S1). qRT-PCR was performed using PowerUP SYBR green (Thermo Fisher Scientific, Waltham, MA, USA) according to manufacturer′s instructions. To calculate differences in RNA expression we used the DDCT method versus *18S rRNA*. Statistical analysis comparison between rSARS-CoV-2 U76G against rSARS-CoV-2 WT 8 h as a control was performed using two-sided unpaired Student′s *t*-test with Prism 7 (GraphPad Software Inc, San Diego, CA, USA).

### 2.20. Covalent RNA Immunoprecipitation Sequencing (cRIP-Seq)

Covalent RNA immunoprecipitation sequencing (cRIP-seq) was performed as previously reported [40]. Briefly, 2.4 × 10^6^ A549 ACE2 cells were infected with rSARS-CoV-2 WT or rSARS-CoV-2 U76G at MOI 0.1 PFU/cell. At 48 hpi, culture media was removed, cells were rinsed and scraped in cold PBS. Following centrifugation (200× *g*, 8 min, 4 °C), the supernatant was completely removed and the cell pellet lysed in 2x Co-IP buffer (100 mM Tris-HCl pH 7.4, 300 mM NaCl, 2% (*v*/*v*) IGEPAL CA-630, 1% sodium deoxycholate, 0.5 mM TCEP, EDTA-free Protease Inhibitor Cocktail (Sigma-Aldrich, St. Louis, MO, USA). After incubation for 30 min at room temperature, an equal volume of water was added and lysis was completed by sonication (2 kJ with 10% amplitude, 0.7 s on/2.3 s off). Fresh lysates were immediately used for immunoprecipitation (IP) and sequencing library preparation as described in cRIP-seq method [40]. Briefly, limited RNase digestion with RNase I was used to trim unprotected RNA followed by IP with Anti-NSP9 antibody (cat. GTX135732-100, GeneTex, Irvine, CA, USA). Afterwards, SDS-PAGE was applied to separate IP and size-matched input (SMI) samples followed by transfer to a nitrocellulose membrane. The expected size range was excised and Proteinase K (New England Biolabs, Ipswich, MA, USA) was employed to release protein-bound RNA. After conversion of IP and SMI RNA into cDNA libraries and amplification with 15 PCR cycles, and sequenced with a Illumina NextSeq instrument(Illumina, San Diego, CA, USA) with a read length of 2 × 40 nucleotides. 

### 2.21. cRIP-Seq Analysis

cRIP paired-end reads were adapter- and quality trimmed using cutadapt (v1.18) [41] Reads with a total length less than 18 nt were discarded. A custom java program was applied that simultaneously identified and clipped the remaining unique molecular identifier (UMI) associated with each read pair. The trimmed reads were aligned to the genomes of *Chlorocebus sabaeus* (Ensembl release 111) genome and SARS-CoV-2 (NC_045512.2, GenBank: MN908947.3) using STAR (v2.7.10a) [42] with the parameters—outFilterScoreMinOverLread 0—outFilterMatchNminOverLread 0—outFilterMatchNmin 0—alignSoftClipAtReferenceEnds No—alignSJoverhangMin 8—alignSJDBoverhangMin 1—outFilterMismatchNoverLmax 0.04—scoreDelOpen−1—alignIntronMin 20—alignIntronMax 3000—alignMatesGapMax 3000—alignEndsType EndToEnd. PCR duplicates were removed with the UMI-aware deduplication functionality of Picard′s MarkDuplicates. The resulting aligned reads were separated based on their strand orientation. Regions with enriched protein binding were identified for each strand with MACS2 [43] using the parameters -g 29903 -s 31 --keep-dup all --nomodel --d-min 25 --summits --scale-to small --shift 25 --nolambda --extsize 5 --max-gap 20 --min-length 5. The identified MACS2 peaks were additionally filtered by calculating the enrichment of strand specific reads within each peak over all remaining strand specific mapped reads between IP and SMI. A statistically significant enrichment relative to SMI control was calculated by a one-sided Fisher′s exact test. The resulting *p* values were corrected with the Benjamini-Yekutieli [44] procedure and only peaks with an adjusted *p* value < 0.05 were considered for further downstream analysis.

Crosslinking sites were defined as the first nucleotide in 5′-direction at the 5′-end of a R2 read that overlaps a significantly enriched peak. Coverage of each crosslinking site in IP and SMI was calculated as the number of R2 reads sharing the same 5′-end. Only crosslinking sites in wildtype samples with at least 20 reads of coverage in IP were included in further analyses. To calculate the enrichment of IP over SMI or wildtype over mutant, Fisher′s exact test was applied in the same manner as described for identifying enriched peaks in IP over SMI. The resulting *p* values were corrected using the Benjamini–Yekutieli procedure. Adjusted *p* values below a threshold of 0.05 were considered statistically significant.

### 2.22. Microscopy

Because SARS-CoV-2 is considered as a BioSafety Level 3 (BSL3) pathogen, all the images of live cells provided in this study were performed inside the BSL3 laboratory using a Revolve R4 microscope (ECHO, San Diego, CA, USA).

### 2.23. Figure Design and Generation

All figures presented in this work were designed and assembled using web-based platform Biorender (BioRender, Toronto, ON, Canada).

## 3. Results

### 3.1. Dividing and Rebuilding a Long Viral Genome

The 30 kb genome of SARS-CoV-2 is among the largest of any known RNA viruses, presenting significant technical challenges for its handling and genetic manipulation [16,45,46,47]. Therefore, as a first step, we decided to divide the genome into smaller regions that could be handled and propagated in plasmids. Bacterial plasmids ensure the safe storage of the SARS-CoV-2 genome *E. coli*, without the need for specialized protocols, and can be manipulated using well-established mutagenesis methodologies [39]. This approach also facilitates the assembly of the full genome SARS-CoV-2 and shuffling of mutations within different fragments, potentially speeding the generation of mutant recombinant viruses.

Six fragments ranging from 6.2 to 3.2 kb were amplified using as a template the SARS-CoV-2 isolate Wuhan-Hu-1 (GenBank MN996528.1 from pCC1BAC-HIS3-SARS-CoV-2). An extra fragment containing a Turbo GFP reporter sequence instead of the ORF7, was amplified using the YAC pCC1BAC-HIS3-SARS-CoV-2 GFP (BioProject: PRJNA615319; BioSample: SAMN14450690; Sample name: GFP-2_rSARS-CoV-2). One fragment (3.5 kb) was inserted into a high copy number plasmid (pUC19) meanwhile the reminding six (6.2 to 3.2 kb) were cloned into a low copy number plasmid (pUA66). The rationale for employing a low-copy-number vector arose from the observed instability of viral sequences in the high-copy-number vector, characterized by recurrent recombination events, bacterial sequence insertions, and loss of the viral sequence from the plasmid. A cytomegalovirus (CMV) promoter (598 bp) was added to the upstream region of the 5′ UTR in fragment F1, replacing the previous T7 promoter. This new promoter enables transcription of the viral genome by cellular RNA polymerase II in the nucleus. A linker sequence was also incorporated after the 3′ UTR in plasmids F6 WT and F6 GFP. The linker comprises three distinct elements: (1) a hepatitis delta virus ribozyme (HDVr) to produce a specific 3′ end in the viral RNA, (2) a simian virus 40 PolyA signal (SV40 polyA) to enhance transcription termination and promote RNA stability, and (3) a 364 bp spacer sequence to create an intermediate area between the extremes of the viral genome during the circularization assembly [38] (Supplementary Figure S1A). After successfully cloning all fragments, we sought to verify the authenticity of the SARS-CoV-2 sequence. Apart from a silent point mutation at position C28103A located in Fragment 6 WT, our sequence was identical to the original genome of SARS-CoV-2 isolate Wuhan-Hu-1.

**Figure 2:**
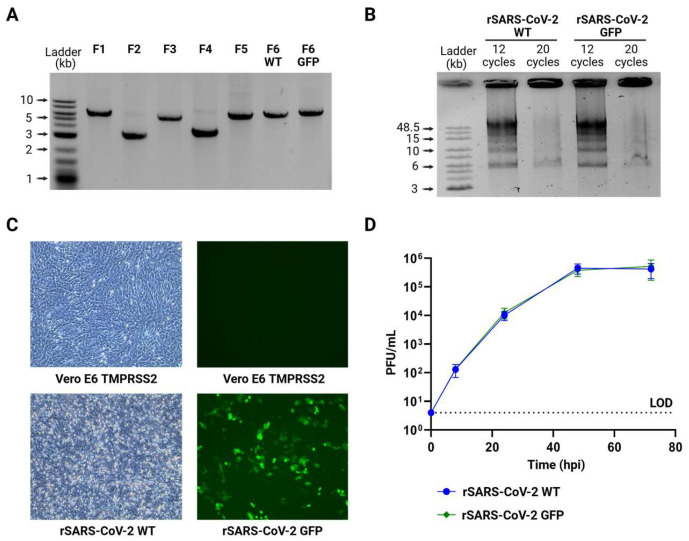
Rescue and characterization of rSARS-CoV-2. (**A**) PCR products of 7 fragments of SARS-CoV-2, including the fragment for the GFP reporter, using bacterial plasmids as template. (**B**) DNA fragments are assembled to produce the 30 kb SARS-CoV-2 DNA, 2 different conditions with different number of assembly cycles were tested. (**C**) Infection using recombinant SARS-CoV-2. After DNA transfection, supernatants were collected and used to infect a monolayer of Vero E6 TMPRSS2, after 2 days cells present CPE for SARS-CoV-2 WT or express GFP for the recombinant SARS-CoV-2 GFP. (**D**) Comparison of growth kinetics of rSARS-CoV-2 WT and rSARS-CoV-2 GFP in Vero E6 TMPRSS2 cells infected at a MOI0.01 PFU/cell over a 72 h time course. LOD: Limit of detection. *n* = 2 independent experiments with two replicates each. The graphic shows mean values ± SD. Viral titers were not significantly different at any time-point by two-sided unpaired Student′s *t*-test.

We chose a PCR-based strategy for the reassembly of the full SARS-CoV-2 genome, which has been previously described for different RNA viruses [24,35,36,38,48]. This method relies on generating SARS-CoV-2 genome fragments with a 20 bp overlap between adjacent fragments, enabling their assembly during PCR. After amplifying and purifying the individual fragments (Figure 2A), each of them was mixed in an equimolar amount of 0.1 pMol. During temperature cycling, in the presence of a high-fidelity DNA polymerase and dNTPs, complementary fragments anneal based on the 20 bp overlap, facilitating the formation of a complete circular genome. To optimize the assembly, we tested two cycling conditions: (1) initial denaturation at 98 °C for 30 s, 12 cycles of 10 s at 98 °C, 20 s at 55 °C and 10 min at 68 °C, followed by a final elongation for 12 min at 68 °C. (2) initial denaturation at 98 °C for 2 min, 20 cycles of 10 s at 98 °C, 15 s at 55 °C and 25 min at 68 °C, and a final elongation for 25 min at 68 °C. Only the first cycling condition successfully produced the full 30 kb genome assembly (Figure 2B). Considering that the primary discrepancy between the two assembly protocols lay in the number of cycles employed (12 and 20 cycles), and recognizing that the optimal condition was achieved with fewer cycles (12 cycles), we proceeded to refine the assembly reaction by evaluating 6 and 9 cycles. However, these conditions yielded incomplete assemblies, evidenced by the presence of multiple bands indicating intermediate products and unused initial fragments (Supplementary Figure S1B,C).

Taking into account all the aforementioned factors, we opted for a PCR-based assembly protocol with an initial denaturation at 98 °C for 30 s, 12 cycles of 10 s at 98 °C, 20 s at 55 °C and 10 min at 68 °C, and a final elongation for 12 min at 68 °C, as the primary strategy for assembling complete SARS-CoV-2 genomes.

### 3.2. Rescue of Recombinant SARS-CoV-2

Unpurified SARS-CoV-2 GFP DNA assembly alongside an expression plasmid coding for N protein was transfected into HEK293T cells overexpressing angiotensin-converting enzyme 2 (HEK293T ACE2) [18,32,34]. HEK293T cells are well-suited for the uptake of external genetic material, and ACE2 expression enhances their susceptibility to SARS-CoV-2 infection [49,50]. To rescue rSARS-CoV-2 GFP, we tested a range of transfection methods, including electroporation, nucleofection, and lipid-based reagents such as Lipofectamine 2000, Lipofectamine 3000, TransIT 2020, TransIT LT1, and polyethylenimine (PEI). Then, 24 h after transfection, HEK293 ACE2 cells were detached with trypsin and seeded onto a monolayer of Vero E6 cells overexpressing transmembrane serine protease 2 (Vero E6 TMPRSS2), which are highly susceptible and permissive to infection, serving as a strategy to enhance the isolation of rSARS-CoV-2 [50,51,52]. We examined GFP expression, as a marker of rescue, every 24 h for 10 days in a BSL3 facility. Remarkably the condition that yielded infectious rSARS-CoV-2 GFP most consistently and cost-effectively was when HEK293T ACE2 cells were transfected using a low-cost reagent, PEI (Supplementary Figure S2). Further optimizations were conducted using varying amounts of DNA combined with different DNA: PEI ratios. Ultimately, the optimal rescue condition was found to be the transfection of 50 µL of SARS-CoV-2 GFP DNA assembly mixed with 200 ng of N plasmid and 28.8 µL of PEI (Supplementary Figure S2).

Using this approach, we successfully obtained infectious rSARS-CoV-2 WT and rSARS-CoV-2 GFP, which induced cytopathic effects (CPE) or displayed GFP expression, respectively (Figure 2C). Titers of 2.9 × 10^5^ PFU/mL for rSARS-CoV-2 WT and 2.5 × 10^5^ PFU/mL for rSARS-CoV-2 GFP were achieved after a single passage, and further analysis revealed that plaque morphology and size were similar for both viruses (Supplementary Figure S3). Next, Vero E6 TMPRSS2 cells were infected separately with each virus at a MOI of 0.01 PFU/cell, and supernatants were collected at 8, 24, 48 and 72 h post infection (hpi). Growth kinetics of both recombinant viruses were not statistically different demonstrating that the rescue of SARS-CoV-2 was successful and that the substitution of ORF7 for reporter GFP did not affect the replication of SARS-CoV-2 in Vero E6 TMPRSS2 cells during the period evaluated (Figure 2D).

In conclusion, we rescued rSARS-CoV-2 by transfecting SARS-CoV-2 PCR-based DNA assembly along with an N expression plasmid into a co-culture of HEK293T ACE2 and VERO E6 TMPRSS2 cells using PEI, a cost-effective transfection reagent. The successful rescue using PEI with unpurified PCR product highlights the high yield and robustness of the assembly reaction, as it does not require specialized or high-cost transfection reagents to achieve infectious titers.

### 3.3. Analysis of the Full Genome of Recombinant SARS-CoV-2 Using Nanopore Sequencing

**Figure 3:**
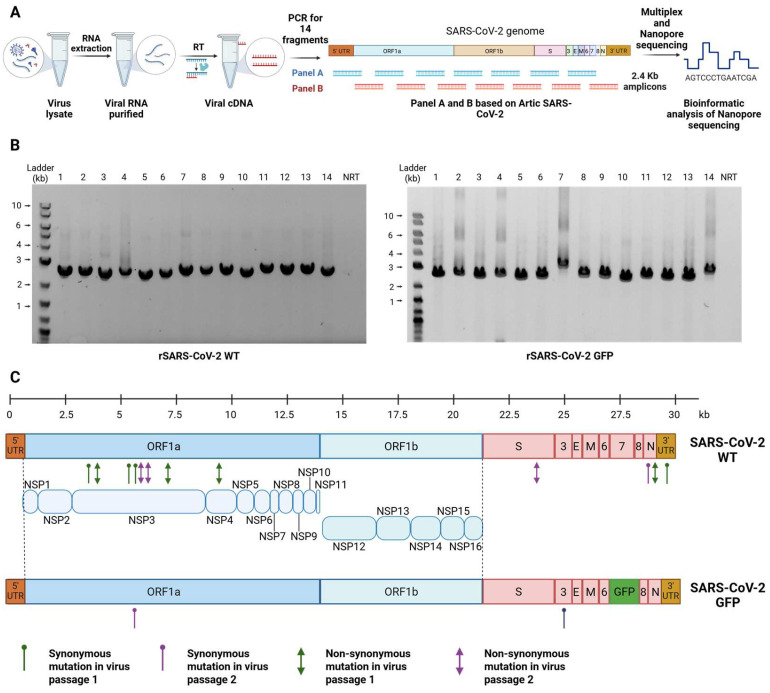
Sequencing of rSARS-CoV-2. (**A**) Oxford Nanopore DNA sequencing workflow for rSARS-CoV-2. (**B**) DNA fragments of SARS-CoV-2 for Nanopore sequencing. An amount of 14 amplicons of 2.4 kb covering the full genome of rSARS-CoV-2 WT (left panel) and rSARS-CoV-2 GFP (right panel) were generated by RT-PCR of RNA extracted from supernatant of Vero E6 TMPRSS2 cells at 72 hpi. (**C**) Schematic representation of rSARS-CoV-2 WT and rSARS-CoV-2 GFP genome. Synonymous (hairpins) and non-synonymous (arrows) mutations of each virus are indicated, specifying those mutations present in two different passages (passage 1 = green and passage 2 = purple).

To assess the accuracy of the RGS and the stability of the genome after passage, we sequenced recombinant viruses after two passages using Oxford Nanopore Sequencing (Figure 3A).

RNA from the supernatants of passage 1 and passage 2 of rSARS-CoV-2 WT and rSARS-CoV-2 GFP grown in Vero E6 TMPRSS2 cells were isolated, reverse transcribed, and amplified into overlapping DNA amplicons. We generated fourteen 2.4 kb amplicons using two panels of primers based on the Artic sequencing protocol for SARS-CoV-2 (Figure 3B). Each of these fourteen amplicons was purified, pooled, multiplexed, barcoded, and sequenced using an Oxford Nanopore MinION device.

The sequences of all recombinant viruses were compared to the reference sequences. Mutations with a frequency exceeding 50% were examined, revealing that the initial passage of the four independently rescued rSARS-CoV-2 WT had a nucleotide accuracy of 99.9933%. The second passage of rSARS-CoV-2 WT maintained a nucleotide accuracy of 99.9866%. Similarly, both the first and second passages of rSARS-CoV-2 GFP showed high-fidelity rescue, with a nucleotide accuracy of 99.9967% (Table 1). At the amino acid level, we identified four non-synonymous mutations (P23L and A1527V in NSP3, I382V in NSP4 and T71M in N) in one of the four independently rescued rSARS-CoV-2 WT during the first passage, resulting in an amino acid fidelity of 99.959%. The remaining three rescued rSARS-CoV-2 WT did not exhibit any non-synonymous mutations in the first passage. In the second passage, three additional mutations arose (T1180S and A1305G in NSP3 and A701T in S), although the amino acid accuracy remained high at 99.969%. On the other hand, only synonymous mutations were detected in rSARS-CoV-2 GFP in both passages (Figure 3C and Supplementary Table S2).

Collectively, our RGS for SARS-CoV-2 demonstrated a rescue accuracy exceeding 99.9% for both nucleotide and amino acid sequences. Moreover, the system enables the rescue of stable rSARS-CoV-2, as evidenced by the minimal mutations observed between passages 1 and 2.

### 3.4. Generation of SARS-CoV-2 Spike Mutants

The transmembrane spike (S) protein is responsible for SARS-CoV-2 entry into the host cell by binding to the ACE2 receptor, initiating the infection process. The S protein region exhibits the highest number of non-synonymous mutations in the entire SARS-CoV-2 genome [7,53,54]. Spike mutations are highly relevant since they are responsible for influencing the virus host range, tissue tropism, antibody escape and pathogenesis. Although multiple studies have evaluated the impact of some of these mutations, most experiments have been conducted using only purified proteins or pseudoviruses expressing the S protein. As a result, these studies may not fully account for the presence of other factors or cell-virus interactions that could influence the natural course of infection [55,56,57,58,59,60,61,62].

**Figure 4:**
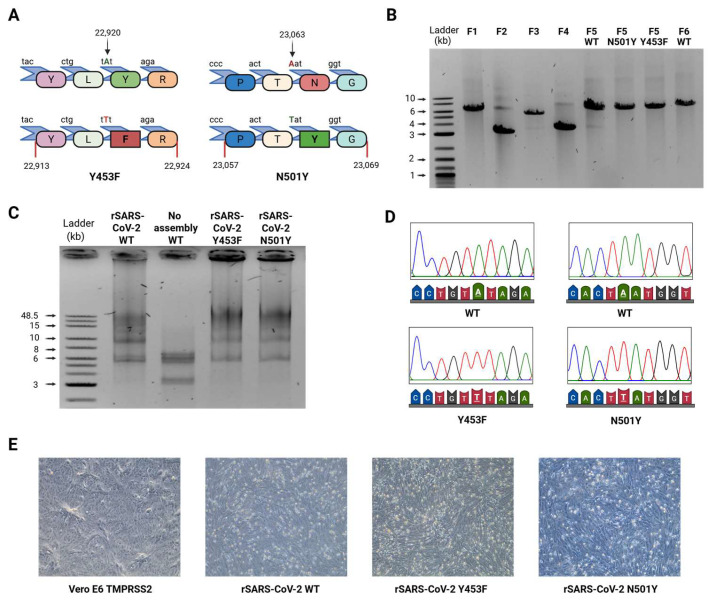
Rescue of rSARS-CoV-2 RBD spike mutants. (**A**) Visual representation of RBD spike mutants. Mutation Y453F and N501Y mutations were introduced in fragment 5, at positions 23,063 and 22,920 of SARS-CoV-2 genome. In both mutations, a single nucleotide was changed in order to alter the amino acid sequence. (**B**) PCR products of 8 fragments of SARS-CoV-2, including the fragment 5 with Y453F and N501Y mutation. (**C**) Assembly of complete DNA SARS-CoV-2. An equimolar mixture of 6 DNA fragments is assembled in the 30 kb SARS-CoV-2 genome, in order to generate different recombinant SARS-CoV-2. A control including all the fragments but without DNA polymerase was performed. (**D**) Sanger sequencing of SARS-CoV-2 RBD spike mutants. Supernatant of rSARS-CoV-2 WT, rSARS-CoV-2 Y453F and rSARS-CoV-2 N501Y infected cells were subjected to Sanger sequencing, showing the desired mutations compared to rSARS CoV-2 WT. (**E**) Infection using recombinant SARS-CoV-2 RBD spike mutants. After DNA transfection, supernatants were collected to infect a monolayer of Vero E6 TMPRSS2 cells, after 2 days CPE was observed for rSARS-CoV-2 WT, rSARS-CoV-2 Y453F and rSARS-CoV-2 N501Y.

Considering all the above, we generated two recombinant viruses containing either the N501Y spike mutation (present in the Alpha, Beta, Gamma and Omicron variants) and Y453F spike mutation (found in the Cluster 5 variant). These mutations, located directly at the interface between the receptor-binding domain (RBD) of the S protein and ACE2, have been linked to increased ACE2-binding affinity [7,58]. First, plasmid F5 was modified by site-directed mutagenesis [39] to introduce the A22920T and A23053T nucleotide exchanges, corresponding to the Y453F and N501Y mutations (Figure 4A). The genome DNA assembly for both mutants was performed under the conditions previously described (Figure 4B,C). We obtained preparations for each of the recombinant viruses with titers of 3.4 x10^5^ PFU/mL for rSARS-CoV-2 Y453F and 5.0 × 10^5^ PFU/mL for rSARS-CoV-2 N501Y, and confirmed the successful introduction of each mutation in the four recombinant viruses by Sanger sequencing (Figure 4D). Similarly, to the previously rescued rSARS-CoV-2 WT, CPE was observed (Figure 4E) and plaque morphology and size were comparable across all viruses (Supplementary Figure S3).

### 3.5. Biological Impact of rSARS-CoV-2 Spike Mutants

ACE2 has been identified as the main entry point for several coronaviruses, including SARS-CoV, MERS-CoV, and SARS-CoV-2 [1,52,63,64]. Given the association between the RBD spike mutations Y453F and N501Y with enhanced ACE2 binding affinity, improved viral fitness, higher transmission rates, and increased viral load [7,9,57,58,65], we investigated their impact on ACE2 binding affinity, replication kinetics, and competition assays. For this analysis, two different cell lines were used: Vero E6 TMPRSS2 and A549 ACE2. Vero E6 cells are epithelial kidney cells that are susceptible and permissive for SARS-CoV-2 infection due to the presence of ACE2 receptor on their membrane [52,66]. In contrast, A549 cells are epithelial lung cells that are susceptible to SARS-CoV-2 infection but poorly permissive due to the absence of ACE2 receptor [50,52]. However, overexpression of ACE2 in A549 cells renders them both susceptible and permissive to SARS-CoV-2 infection [67,68]. Since we did not have precise information on the levels of ACE2 receptor in those cell lines, we first examined expression levels by RT-qPCR and Western blot. In A549 ACE2 cells, we observed *ACE2* mRNA expression six orders of magnitude higher than that observed in Vero E6 TMPRSS2 cells (Supplementary Figure S4A). Despite the variation in ACE2 mRNA expression, no significant difference was found in ACE2 protein levels between Vero E6 TMPRSS2 and A549 ACE2 cells (Supplementary Figure S4B). Notably, Vero E6 TMPRSS2 cells express a monkey ACE2 (mACE2) whereas A549 cells express a human ACE2 (hACE2). We therefore performed a protein alignment to compare these proteins and assess their identity. Bioinformatics tools revealed a 94.66% identity between the two proteins. Importantly, all amino acids involved in interactions with spike residues 501 and 453 were completely identical between the two proteins (Supplementary Figure S4C,D) [50,55,69,70,71].

**Figure 5:**
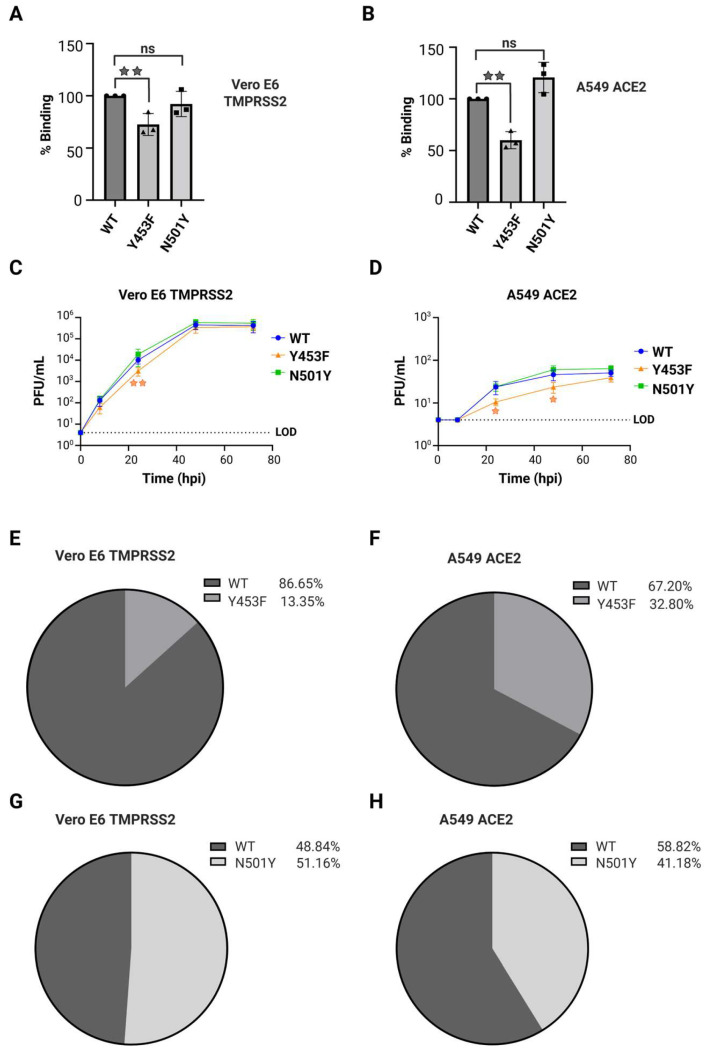
Biological characterization of rSARS-CoV-2 RBD spike mutants. (**A**,**B**) Binding assay for SARS-CoV-2 spike mutants. Vero E6 TMPRSS2 cells or A549 ACE2 were incubated with rSARS-CoV-2 WT, rSARS-CoV-2 Y453F or rSARS-CoV-2 N501Y at 4 °C to evaluate the interaction between the spike and ACE2 receptor. Samples were evaluated by qPCR for RdRp and compared to rSARS-CoV-2 WT in (**A**) Vero E6 TMPRSS2 at a MOI 0.1 PFU/cell and (**B**) A549 ACE2 at a MOI 0.1 PFU/cell. Quantification relative to 18S rRNA. n = 3 independent experiments with two replicates each. The graphic shows mean values ± SD. Asterisks above the bars indicate the degree of significance compared to the control condition (* = *p* < 0.05, ** = *p* < 0.01, ns = not significant by one-way ANOVA). (**C**,**D**) Replication kinetics of rSARS-CoV-2 N501Y and Y453F. Comparison of growth kinetics of rSARS-CoV-2 RBD spike mutant to rSARS-CoV-2 WT in (**C**) Vero E6 TMPRSS2 or (**D**) A549 ACE2 cells infected at a MOI0.01 PFU/cell over a 3-day time course. LOD: Limit of detection. *n* = 2 independent experiments with two replicates each. The graphic shows mean values ± SD. Asterisks indicate the degree of significance compared to the rSARS-CoV-2 WT control (* = *p* < 0.05, ** = *p* < 0.01, ns = not significant by two-sided unpaired Student′s *t*-test). (**E**,**F**) Competition assay for rSARS-CoV-2 Y453F. (**D**) Vero E6 TMPRSS2 and (**E**) A549 ACE2 cells were infected with rSARS-CoV-2 WT plus rSARS-CoV-2 Y453F at a MOI 0.1 PFU/cell. After 48 hpi, RNA from supernatant infected with was collected and processed for sequencing by Oxford Nanopore. Percentage of reads corresponding to the respective rSARS-CoV-2 mutant sequence. *n* = 2 independent experiment with two duplicates. (**G**,**H**) Competition assay for rSARS-CoV-2 N501Y. (**G**) Vero E6 TMPRSS2 and (**H**) A549 ACE2 cells were infected with rSARS-CoV-2 WT plus rSARS-CoV-2 N501Y at a MOI 0.1 PFU/cell. After 48 hpi, RNA from supernatant infected with were collected and processed for sequencing by Oxford Nanopore. Percentage of reads corresponding to the respective rSARS-CoV-2 mutant sequence. n = 2 independent experiment with two duplicates.

After confirming similar ACE2 protein levels and a high degree of protein identity between the two cell lines, we evaluated the binding affinities of rSARS-CoV-2 spike mutants. Binding assays were carried out by incubating a monolayer of cells with virus at 4 °C, during which the biochemical interaction between S protein and ACE2 receptor can occur, but viral entry is inhibited [72,73]. Vero E6 TMPRSS2 and A549 ACE2 cell lines were separately incubated with recombinant SARS-CoV-2 viruses: rSARS-CoV-2 WT, rSARS-CoV-2 Y453F or rSARS-CoV-2 N501Y using a MOI of 0.1 PFU/cell at 4 °C for 1 h. After incubation, unbound virus particles were washed away with ice-cold PBS, while the viruses that had interacted with ACE2 remained attached. The amount of virus attached to the cell monolayer was quantified via RT-qPCR targeting *RdRp* (Supplementary Figure S5A). Binding efficiency of each spike mutant was compared to rSARS-CoV-2 WT. Interestingly, rSARS-CoV-2 Y453F exhibited reduced binding efficiency relative to rSARS-CoV-2 WT in both cell lines (Figure 5A,B). Meanwhile, rSARS-CoV-2 N501Y showed no significant difference in binding efficiency compared to rSARS-CoV-2 WT in either cell line (Figure 5A,B). Additionally, no significant differences in binding were observed between the two cell lines for any of the recombinant viruses tested (Supplementary Figure S5B–D).

Next, we evaluated the replication kinetics of spike mutant viruses in Vero E6 TMPRSS2 and A549 ACE2 cells. Both cell lines were separately infected with rSARS-CoV-2 WT, rSARS-CoV-2 Y453F or rSARS-CoV-2 N501Y at a MOI of 0.01 PFU/mL and samples were collected at 8, 24, 48 and 72 hpi for titration by plaque assay (Figure 5C,D). We consistently observed lower viral titers at each time point in A549 ACE2 compared to Vero E6 TMPRSS2 cells, indicating that A549 ACE2 cells have lower permissibility to SARS-CoV-2 compared to Vero E6 TMPRSS2 cells, as previously reported [50,52,74]. Additionally, rSARS-CoV-2 Y453F showed slower replication, with lower titers at 24 hpi in Vero E6 TMPRSS2 cells and at both 24 and 48 hpi in A549 ACE2 cells. However, by 72 hpi the titers of the Y453F mutant reached levels comparable to those of rSARS-CoV-2 WT. In contrast, rSARS-CoV-2 N501Y displayed similar replication kinetics to rSARS-CoV-2 WT in both cell lines (Figure 5C,D).

To further assess whether the introduced RBD spike mutants conferred a growth advantage or disadvantage, we conducted competition assays in which Vero E6 TMPRSS2 or A549 ACE2 cells were co-infected at a MOI 0.1 PFU/cell with rSARS-CoV-2 WT plus rSARS-CoV-2 Y453F or rSARS-CoV-2 N501Y. As controls, we also infected both cell lines with rSARS-CoV-2 WT, rSARS-CoV-2 Y453F and rSARS-CoV-2 N501Y separately. At 48 hpi, RNA was extracted from supernatants and subsequently prepared for Oxford Nanopore sequencing, as previously described (Supplementary Figure S6A). In cells infected with only one virus, we observed a correlation of over 90% between each recombinant virus and the corresponding nucleotide position analyzed (Supplementary Figure S6B,C).

Result from the competition assays showed that rSARS-CoV-2 WT virus outperformed the rSARS-CoV-2 Y453F mutant in both cell lines. In Vero E6 TMPRSS2 cells, WT accounted for 86.65% of the distribution compared to 13.35% for Y453F (Figure 5E). Similarly, in A549 ACE2 cells, rSARS-CoV-2 WT had a presence of 67.20% versus 32.80% for rSARS-CoV-2 Y453F (Figure 5F). Meanwhile, rSARS-CoV-2 N501Y and rSARS-CoV-2 WT exhibited similar distributions during competition assays in Vero E6 TMPRSS2 cells, with rSARS-CoV-2 N501Y accounting for 51.16% and rSARS-CoV-2 WT for 48.84%, indicating no significant advantage for the mutant (Figure 5G). However, in A549 ACE2 cells, the competition assay showed that rSARS-CoV-2 WT virus replicated slightly better than rSARS-CoV-2 N501Y, with 58.82% for WT and 41.17% for N501Y, although this difference was not statistically significant (Figure 5H).

Taken together, our results revealed distinct phenotypes for the N501Y and Y453F mutants regarding ACE2 binding and viral infectivity. The rSARS-CoV-2 Y453F mutant exhibited decreased binding compared to the rSARS-CoV-2 WT virus, likely contributing to its slower replication kinetics and lower fitness in competition assays. In contrast, the rSARS-CoV-2 N501Y mutant showed comparable binding efficiency, replication kinetics and similar fitness in competition assays to the rSARS-CoV-2 WT in both cell lines.

### 3.6. A Putative Role for Anti-Leader Sequence in SARS-CoV-2 Replication

During the replication cycle of SARS-CoV-2, a set of subgenomic RNAs (sgRNAs) are generated to produce viral structural and non-structural proteins. sgRNAs synthesis starts at the 3′ end of the genome and employs a discontinuous transcription mechanism to generate negative-sense sgRNAs (-sgRNAs), which are later transcribed into positive-sense subgenomic RNAs (+sgRNAs) [3,5]. Template-switching is proposed as the main mechanism underlying discontinuous transcription. This process requires a 50 bp leader transcription regulatory sequence (TRS-L) in the 5′ UTR and eight body transcription regulatory sequences (TRS-B) distributed across the genome upstream of each viral subgenomic mRNA (sgmRNA). Although the precise molecular mechanism remains unclear, base-pairing interactions between the TRS-L and the TRS-B upstream of each sgmRNA likely facilitate the fusion of the leader and body sequences. This fusion generates at least eight sgRNAs, which vary in length depending on the TRS-B location but share identical 5′ and 3′ ends [2,3,5,68,75].

**Figure 6:**
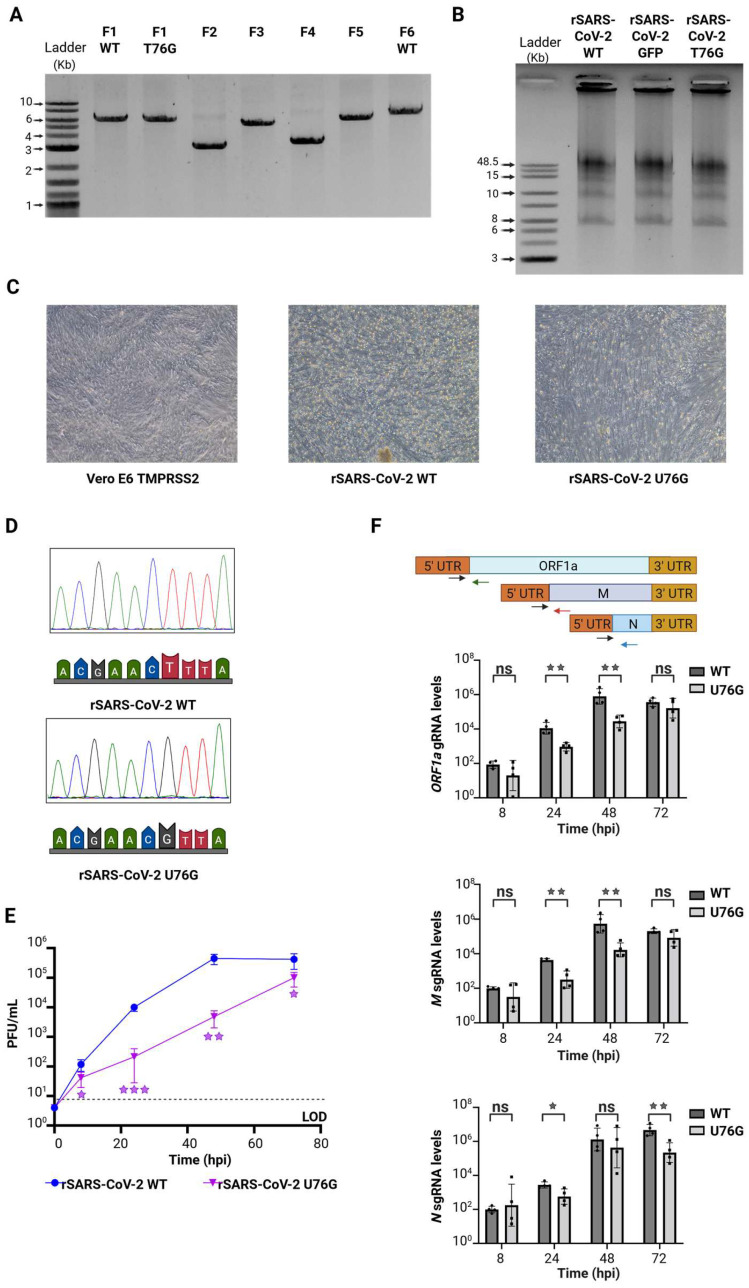
Rescue and characterization of rSARS-CoV-2 5′UTR mutant. (**A**) PCR products of 7 fragments of SARS-CoV-2, including fragment 1 with T76G mutation. (**B**) Assembly of complete DNA SARS-CoV-2. An equimolar mixture of 6 DNA fragments is assembled in the 30 kb SARS-CoV-2 genome, in order to generate different rSARS-CoV-2. (**C**) Infection using recombinant SARS-CoV-2 U76G mutant. After DNA transfection, supernatants were collected to infect a monolayer of Vero E6 TMPRSS2 cells, after 2 days CPE was showed for rSARS-CoV-2 WT and rSARS-CoV-2 U76G. (**D**) Sanger sequencing of rSARS-CoV-2 5′UTR mutant. Supernatant of rSARS-CoV-2 WT and rSARS-CoV-2 U76G infected cells were subjected to Sanger sequencing, showing the desired mutation compared to rSARS CoV-2 WT. (**E**) Comparison of growth kinetics of rSARS-CoV-2 U76G mutant against rSARS-CoV-2 in Vero E6 TMPRSS2 cells infected at a MOI0.01 PFU/cell over 72 h time course. n = 2 independent experiments with two replicates each. The graphic shows mean values ± SD. Asterisks indicate the degree of significance compared to the rSARS-CoV-2 WT or rSARS-CoV-2 GFP control (* = *p* < 0.05, ** = *p* < 0.01, *** = *p* < 0.001, ns = not significant by two-sided unpaired Student′s *t*-test). (**F**) gRNA and sgRNA expression for rSARS-CoV-2 U76G. Upper. Schematic primer design to quantify gRNA and sgRNA expression. Lower. RT-qPCR of ORF1a, M and N RNA levels at 8, 24, 48 and 72 hpi in Vero E6 TMPRSS2 cells infected with rSARS-CoV2 WT or rSARS-CoV-2 U76G. Quantification relative to 18S rRNA. n = 2 independent experiments with two replicates each. The graphic shows mean values ± SD. Asterisks near the bars indicate the degree of significance compared to the rSARS-CoV-2 WT 8 hpi (* = *p* < 0.05, ** = *p* < 0.01, *ns* = not significant by Student′s *t*-test).

The viral protein NSP9 is an RNA-binding protein that is suggested to play a role in RNA priming and capping during transcription, although its primary functions remain unclear [40,68,76,77,78,79,80,81,82]. A recent study using covalent RNA immunoprecipitation (cRIP) demonstrated that NSP9 is covalently attached to the 5′ ends of SARS-CoV-2 RNAs. In addition to the annotated genome ends, cRIP identified an unusual covalent linkage at position 76 in the negative-sense viral RNA [40]. Interestingly, this site is located adjacent to the TRS-L, suggesting that NSP9 may influence discontinuous transcription or template switching. To test this hypothesis, we modified plasmid F1 to induce a T76G point mutation into the plasmid containing the SARS-CoV-2 5′ UTR by site-directed mutagenesis [39]. rSARS-CoV-2 U76G was successfully rescued following the same conditions previously described, producing CPE after 48 hpi in Vero E6 TMPRSS2 cells (Figure 6A–C). Sanger sequencing analysis of the rSARS-CoV-2 T76G virus confirmed the presence of the intended mutation (Figure 6D). Titration of the viral stock via plaque assay indicated rescue efficiencies of 7.0 x10^5^ PFU/mL for rSARS-CoV-2 U76G. Notably, rSARS-CoV-2 U76G displayed smaller plaque size compared to rSARS-CoV-2 WT (Supplementary Figure S3).

We then evaluated the replication kinetics of rSARS-CoV-2 U76G virus alongside rSARS-CoV-2 WT. Using a MOI of 0.01 PFU/cell, we infected Vero E6 TMPRSS2 cells and collected samples at 8, 24, 48, and 72 hpi (Figure 6E). rSARS-CoV-2 U76G showed a striking growth deficiency at each time point evaluated compared to rSARS-CoV-2 WT, with the highest significant difference at 24 hpi. Subsequently, we asked whether the mutation U76G next to the TRS-L could impair the synthesis of gRNA or sgRNAs, potentially explaining the reduced titers observed in the replication kinetic assays. To test this, Vero E6 TMPRSS2 cells were infected at a MOI 0.1 PFU/cell with either rSARS-CoV-2 WT or rSARS-CoV-2 U76G, and samples were collected at 8, 24, 48 and 72 hpi. RNA levels of gRNA and two positive sense sgRNAs were quantified using RT-qPCR with primers targeting the leader-body junction for ORF1a gRNA and the M and N sgRNA junctions [40]. Compared to rSARS-CoV-2 WT, rSARS-CoV-2 U76G exhibited significantly reduced levels of *ORF1ab* gRNA and *M* sgRNA at 24 and 48 hpi. Notably, *N* sgRNA levels were also decreased during rSARS-CoV-2 U76G infection at 24 hpi and at 72 hpi compared to rSARS-CoV-2 WT (Figure 6F).

**Figure 7:**
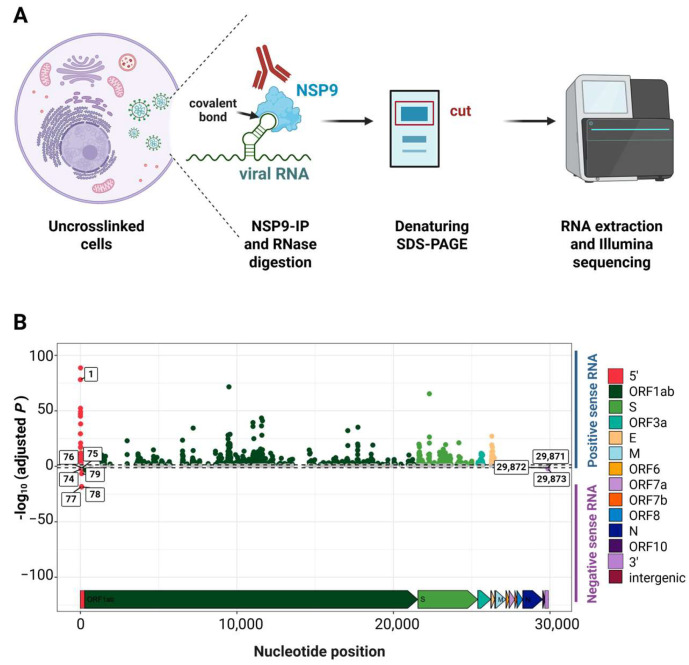
NSP9 binding sites by covalent RNA immunoprecipitation. (**A**) Schematic of covalent RNA immunoprecipitation (cRIP) to map covalent RNA-protein linkages formed in the absence of UV-crosslinking. (**B**) U76G-dependent changes in covalent NSP9-RNA linkages across the SARS-CoV-2 genome in positive and negative-sense RNA. Representative experiment in Vero E6 TMPRSS2 cells (rSARS-CoV-2 WT vs. rSARS-CoV-2 U76G) at 24 hpi is shown. One out of two independent experiments shown.

Given the observed reductions in gRNA and sgRNA levels, along with the proposed role of NSP9 in transcription and its previously identified binding at U76, we next evaluated NSP9 binding at position 76 in the negative-sense viral RNA and other key sites previously reported [40]. Vero E6 TMPRSS2 cells were infected with either rSARS-CoV-2 WT or rSARS-CoV-2 U76G at a MOI of 0.1 PFU/cell, and samples were collected at 24 hpi for covalent RNA immunoprecipitation sequencing (cRIP-seq). Sequence reads covalently linked to NSP9 were analyzed, separating them based on their origin from the positive or negative viral RNA strand, and peaks significantly enriched relative to a size-matched input (SMI) control were identified (Figure 7A).

By analyzing NSP9 binding at the 5′ ends of both positive sense and negative sense viral RNA, we identified a significant reduction in NSP9 binding at position 76 in the negative-sense viral RNA during rSARS-CoV-2 U76G infection compared to rSARS-CoV-2 WT. Additionally, NSP9 binding was observed at positions 74 to 79 in the negative-sense viral RNA in Vero E6 TMPRSS2 cells, sites not previously detected in A549 cells [40]. Notably, NSP9 binding at these additional sites was also reduced during rSARS-CoV-2 U76G infection relative to rSARS-CoV-2 WT (Figure 7B and Supplementary Figure S7).

Overall, our findings show that the U76G mutation in the 5′ UTR of SARS-CoV-2 significantly impairs NSP9 binding to position 76 in the negative-sense viral RNA, as well as at additional sites. Notably, the reduced linkage to positive stranded RNA at nucleotide 1 is consistent with reduced positive stranded RNA priming and synthesis. These reductions correlate with observed deficiencies in gRNA and sgRNA synthesis, diminished growth kinetics and smaller plaque sizes compared to the wild-type virus, suggesting that the binding of NSP9 to these regions impacts viral transcriptional and replication.

## 4. Discussion

RGSs allow the rescue and manipulation of recombinant DNA and RNA viruses, facilitating the study of their biological properties and improving our understanding of emerging viral pathogens. Here, we established a versatile and cost-effective PCR-based reverse genetic system for engineering SARS-CoV-2. We achieved this through three key steps: dividing and cloning the SARS-CoV-2 genome into six stable bacterial plasmids, employing a PCR-based assembly strategy, and conducting low-cost transfection of the assembled genome into mammalian cells. This approach enabled accurate and efficient rescue of rSARS-CoV-2.

Previously, the cloning and storage of long SARS-CoV-2 sequences in bacterial plasmids proved to be unstable [16]. Although yeast artificial chromosomes (YACs) and bacterial artificial chromosomes (BACs) allow for the cloning and manipulation of the entire SARS-CoV-2 genome and mitigate issues of plasmid instability and toxicity, these systems require significant technical expertise and do not support the simultaneous introduction of multiple mutations [17,83]. To address these challenges, we divided the SARS-CoV-2 genome into six fragments, one of the lowest fragment number reported to date, and cloned them into low-copy vectors for propagation in *E. coli* and [17,35,36]. This strategy not only improved storage stability but also facilitated the use of well-established cloning methodologies for precise and simultaneous genetic engineering [39].

Several strategies for reconstituting SARS-CoV-2 from fragments have been described, including in-yeast genome assembly [18], Golden Gate-like assembly [22,32,34] and polymerase extension reaction [35,36] (Supplementary Figure S8). In our hands, Golden Gate-like assembly failed to produce the desired results, primarily yielding incomplete viral genome assemblies and limiting the availability of a complete genome for mammalian cell transfection. Instead, we employed a PCR-based assembly strategy, which aligns genome fragments with 20 bp overlaps to ensure efficient and accurate reconstruction of the SARS-CoV-2 genome. This approach not only resulted in high-yield genome assembly but was also shorter and more straightforward.

In previous PCR-based assembly systems for SARS-CoV-2, introducing a desired mutation involves the generation of an extra fragment [35,36], which could lead to subsequent issues during the assembly process. In our system, mutations are introduced directly into the plasmids without increasing the number of fragments needed for the final assembly. Furthermore, viruses with multiple mutations can be easily generated by combining fragments containing the desired mutations. An alternative PCR-based assembly method has been reported, utilizing DNA fragments generated directly from reverse-transcribed RNA without cloning [36]. While this strategy circumvents plasmid toxicity or instability in bacteria, generating fragments directly via reverse transcription carries the risk of introducing unintended mutations due to the error rate of reverse transcriptase, typically around 10^−4^. In contrast, generating fragments using plasmids as templates and employing a high-fidelity DNA polymerase, with a substantially lower error rate of 10^−6^, minimizes the likelihood of unintended mutations.

Our genome assembly design included key regulatory elements, reported by others, to enable efficient viral RNA transcription directly within mammalian cells. These features include a CMV promoter to drive RNA transcription by RNA polymerase II in the nucleus, an HDV ribozyme to generate the authentic viral 3′ end with a polyA tail, and an SV40 polyA signal to ensure proper transcription termination [24,35,36,38,48]. This streamlined design allowed the assembled genome to be directly transfected into mammalian cells. Although this approach precludes the use of T7-mediated in vitro transcription—thereby preventing the generation of infectious RNA in cell-free reactions—it purposefully bypasses the expensive and complex in vitro RNA production and transfection steps associated with handling large, unstable RNA transcripts [18,22,32,34]. Instead, we demonstrated that transfection with PEI, a low-cost transfection reagent, enabled the rescue of high titers of rSARS-CoV-2 even after just one passage. Recently, a new RGS strategy for SARS-CoV-2, known as Infectious Subgenomic Amplicon (ISA) allowed the rescue of rSARS-CoV-2 by directly transfecting overlapping fragments, thus bypassing the need for assembly [37,84]. However, despite its apparent simplicity, this strategy requires the use of expensive transfection agents and involves a longer rescue time post-transfection, including additional viral passages (Supplementary Figure S9). Nanopore sequencing revealed high-fidelity rescue after 2 passages (~99.99% nucleotide accuracy and 99.97% amino acid accuracy), with only 1 to 4 nucleotide mutations detected across the full genome (Table 1). This fidelity is comparable to or exceeds that of established CPER-based systems, which have been reported to exhibit 3 to 5 nucleotide substitutions in similar rescue experiments. Most observed mutations were transitions (Table S2), consistent with the error profile of polymerases, and their presence at high frequencies suggest their selection during passaging in cell culture, as reported previously in other systems [35,36,85].

We used our RGS to explore the impact of different mutations on SARS-CoV-2 infection. We first focused on the RBD of the S protein due to its ongoing and rapid evolution [7,53,54] and generated two different RBD S protein mutant viruses: rSARS-CoV-2 Y453F and rSARS-CoV-2 N501Y. The mutations, Y453F and N501Y, emerged early in the pandemic and have been reported to enhance the binding of S protein to the ACE2 receptor [7,57,58]. ACE2 has been reported as the principal receptor for some coronaviruses like SARS-CoV and SARS-CoV-2 [1,52,63,64]. The Y453F mutation was first identified in humans with contact to infected minks [7] and is located in the RBD, interacting directly with ACE2 [55,56]. This mutation has been associated with increased binding of the isolated RBD to hACE2 in biophysical [55] and deep mutational screening assays [58]. However, our results showed a significant decrease in the binding of rSARS-CoV-2 Y453F to Vero E6 TMPRSS2 and A549 ACE2 cells compared to rSARS-CoV-2 WT. Moreover, it exhibited slightly slower replication kinetics and reduced fitness during competition assays against rSARS-CoV-2 WT in both cell lines. Consistent with our findings, the Y453F mutation has been reported to enhance binding of S protein primarily to the mink ACE2 receptor [86] and attenuate SARS-CoV-2 replication in human cells [56,87]. Together with the fact that Y453F mutation has disappeared from circulating human SARS-CoV-2 strains, our data support the hypothesis that this mutation represents a mink-specific adaptation that ′spilled back′ to humans.

The second mutation, N501Y, has been widely investigated due to its early emergence and its presence in several variants of concern, including Alpha, Beta, Gamma, and Omicron [7,53,54]. The N501Y mutation has been associated with enhanced fitness, higher transmission, and increased viral load [7,9,57,65]. Although the N501Y mutation reduces neutralization by certain RBD-specific antibodies [7,88,89], most studies agree that its enhanced fitness is due to an increased affinity of the RBD of the S protein for the hACE2 receptor [57,58,59,60,61,62]. In our study, we observed similar cell binding, replication kinetics, and fitness during competition assays of rSARS-CoV-2 N501Y compared to rSARS-CoV-2 WT in both cell lines evaluated. Although our results may appear contradictory to previous reports, most studies showing increased binding of N501Y S protein to hACE2 were conducted through computational modeling or in vitro experiments focusing solely on the RBD domain, rather than in the context of full S protein [59,60,61,62]. To our knowledge, only two studies employing pseudotyped virus particles have characterized the N501Y mutation [56,90]. The first study found no difference in infectivity compared to rSARS-CoV-2 WT during infection of Vero cells [90], while the second reported only a small increase in infectivity of HIV-1 N501Y in HEK ACE2 cells [56]. Additionally, an rSARS-CoV-2 N501Y virus was shown to replicate better than rSARS-CoV-2 WT in vivo (in hamsters) and in human airway epithelial (HAE) cells; however, the fitness difference in Vero E6 cells was not as considerable [57]. This suggests that in cell lines with high ACE2 surface expression, receptor binding may not be the rate-limiting step for viral entry, thereby masking the affinity advantage of N501Y that is observed. Furthermore, this study evaluated the N501Y mutation in the context of the D614G mutant, while our study assessed the N501Y mutant in the context of the ancestral Wuhan strain, which does not contain the D614G mutation that rapidly became dominant in circulating human strains [22,91,92]. Previous studies have highlighted the importance of epistasis in SARS-CoV-2 evolution [57,92]. Therefore, we speculate that the fitness advantage of N501Y may be dependent on the presence of the D614G mutation, and its introduction into the ancestral background (as performed here) yields a neutral phenotype due to the absence of these synergistic effects.

Taken together, our data highlight the complexity of exploring the relative fitness of S protein mutants, and it becomes evident that various approaches and techniques need to be combined to enable meaningful evaluation of viral fitness. Biochemical, biophysical and cell culture-based assays using only the RBD domain of S protein are of fundamental importance but may fail to capture the effects of spike mutations on ACE2 affinity in the context of the full S protein, the interaction with ACE2 receptor, or the presence of co-receptors like NRP1 [91]. Even when mutants are assessed in the context of the authentic virus, characterization studies should also consider the effects of epistasis, a powerful force affecting the evolution of protein sequences [92,93,94], as seen with D614G mutation, which is known to shift the epistatic landscape of the S protein of SARS-CoV-2 [57,70,71].

Last, we explored a protein-interaction site adjacent to the TRS-L in the 5′-UTR of SARS-CoV-2. The viral protein NSP9 has been reported to be NMPylated by NSP12 and this modification has been hypothesized as a first step for priming and capping functions during RNA transcription [40,68,76,79,80,82,95]. Supporting these roles, a recent study reported the covalent binding of NSP9 to the 5′ ends of both positive sense RNA and negative sense RNA of SARS-CoV-2 [40]. Additionally, an interesting third binding site of NSP9 was reported: a single adenine residue located at position 76 of negative sense viral RNA [40]. Due to this nucleotide being located adjacent to the TRS-L, we wondered whether this unexpected position might have relevance in the process of discontinuous transcription or template switching. Upon rescuing the mutant rSARS-CoV-2 U76G, we observed a marked reduction in NSP9 binding to position 76 on the negative-sense RNA through cRIP-seq. This reduced binding was associated with a replication defect compared to rSARS-CoV-2 WT when assessing virus growth kinetics. We also detected lower levels of *ORF1a* gRNA, as well as *M* sgRNA and *N* sgRNA, indicating that the U76G mutation disrupts both gRNA and sgRNA synthesis. Although we did not observe a differential effect on the ratio of gRNA to sgRNA levels that would specifically suggest a role in discontinuous transcription, the mutation U76G and NSP9 binding at this position proved important for efficient transcriptional processes and viral replication.

Furthermore, cRIP-seq analysis identified two additional NSP9 binding sites at positions 77 and 78 on the negative-sense RNA, observed during infection in Vero E6 TMPRSS2 cells but not previously reported in A549 cells [40]. The presence of uracil at all positions (76, 77, and 78) suggests a potential “flexibility” in NSP9′s priming position, which may vary depending on the cell line and warrants further study. Importantly, NSP9 binding to positions 77 and 78 was also diminished in the rSARS-CoV-2 U76G mutant compared to rSARS-CoV-2 WT. These reductions across adjacent positions suggests that the U76G mutation may alter NSP9′s binding dynamics in regions potentially involved in priming and hypothesized to be critical for non-canonical discontinuous transcription [5] or anti-leader synthesis [40]. However, the existence of a short “anti-leader” containing RNA remains speculative and requires further exploration. To our knowledge, no prior studies have explored the effects of a U76G mutation, including its interaction with NSP9 or other viral or cellular proteins. While our cRIP-seq data demonstrate a clear change in covalent binding upon mutation of this region, further research is essential to define the precise structural determinants of this interaction and fully elucidate the mechanisms and implications of this mutation for SARS-CoV-2 replication, as well as NSP9′s broader regulatory role in viral RNA synthesis.

In conclusion, we have developed a low-cost and efficient SARS-CoV-2 reverse-genetics strategy that streamlines the generation of viral mutants and enables rapid studies on vaccine efficacy, immune escape, and complex virus–host interactions.

## Figures and Tables

**Table 1:** Rescue accuracy of SARS-CoV-2.

Virus	Independent Rescue	Passage	% Nucleotide Rescue Accuracy	Number of Nucleotide Mutations	% Amino Acid Rescue Accuracy	Number of Amino Acid Mutations
WT	1	1	99.9967	1	100.0000	0
	2	1	99.9866	4	99.9590	3
	3	1	99.9933	2	100.0000	0
	4	1	99.9967	1	100.0000	0
WT	1	2	99.9866	4	99.9693	3
GFP	1	1	99.9967	1	100.0000	0
GFP	1	2	99.9967	1	100.0000	0

## Data Availability

All data supporting the findings of this study are available within the manuscript and its Supporting Information files. Sequencing data generated in this study is available at accession number PRJEB105095 at the European Nucleotide Archive. Additional information and materials are available from the corresponding author upon reasonable request.

## Supplementary Materials

The following supporting information can be downloaded at: https://www.mdpi.com/article/10.3390/v17121604/s1, **Supplementary Figure S1.** General overview and design of PCR-based assembly for SARS-CoV-2 DNA. (**A**) Schematic representation of SARS-CoV-2 WT and rSARS-CoV-2 GFP. Some modifications were introduced into the SARS-CoV-2 genome. First, a CMV promoter was added to the upstream region of the 5’ UTR in fragment F1. Next, a linker sequence was introduced after the 3’ UTR in fragments F6 WT and F6 GFP consisting of: (1) Hepatitis delta virus ribozyme: cleavage of the phosphodiester bound for a precise 3’ end of the viral RNA; (2) SV40 Poly a terminator: ending of transcription to avoid the generation of concatemeric RNA and (3) a Spacer sequence: 350 bp to create an intermediate area between SV40 and the CMV transcription promoter during the circularization assembly. (**B**) DNA fragments of SARS-CoV-2. PCR products of 7 fragments of SARS-CoV-2, including the fragment for the GFP reporter, using bacterial plasmids as template. (**C**) Optimization of assembly for SARS-CoV-2 DNA. Three different assembly conditions (12, 9 and 6 polymerase extension cycles) were tested using an equimolar mixture of 6 DNA fragments to assemble the 30 kb SARS-CoV-2 genome.; **Supplementary Figure S2.** Gene delivery for rescue of rSARS-CoV-2. SARS-CoV-2 DNA assembly and N plasmid were nucleofected, electroporated or lipofected using different transfection reagents into HEK293T ACE2 cells. After 24 h, cells were trypsinized and seeded over a monolayer Vero E6 TMPRSS2 cells. After 10 days, expression of GFP was evaluated as a sign of rescue of rSARS-CoV-2 GFP.; **Supplementary Figure S3.** Plaque morphology of recombinant SARS-CoV-2. Representative plaque morphology of rSARS-CoV-2 WT, rSARS-CoV-2 GFP, rSARS-CoV-2 N501Y, rSARS-CoV-2 Y453F, and rSARS-CoV-2 U76G in Vero E6 TMPRSS2 at 72 hpi.; **Supplementary Figure S4.** Characterization of ACE2 in mammalian cells. (**A**) RT-qPCR analysis of ACE2 mRNA levels in Vero E6 TMPRSS2 and A549 ACE2 as indicated. Quantification relative to 18S rRNA and Vero E6 TMPRSS2 cells. n  =  2 independent experiments with two replicates each. The graphic shows mean values ± SD. Asterisks indicate the degree of significance compared to Vero E6 TMPRSS2 cells (* = *p* < 0.05, ** = *p* < 0.01, *** = *p* < 0.001, **** = *p* < 0.0001 by two-sided unpaired Student’s *t*-test). (**B**) Western blot analysis of ACE2 in Vero E6 TMPRSS2, A549 ACE2 and A549 cells. Actin serves as control. Quantification of ACE2 protein levels in two replicate experiments is shown on the right. The graphic shows mean values ± SD. Asterisks indicate the degree of significance compared to Vero E6 TMPRSS2 cells (* = *p* < 0.05, ** = *p* < 0.01, *** = *p* < 0.001, **** = *p* < 0.0001, *ns* = not significant by two-sided unpaired Student’s *t*-test). (**C**) Comparison of ACE2 protein identity. Protein sequences for hACE2 and mACE2 were compared using UniProt and NIH alignment bioinformatics tools. The percentage inside the box represents the identity between the proteins. (**D**) Comparison of amino acid residues critical for binding with the residues Y453 and N501 in the S protein. Comparison of hACE2 and mACE2 protein sequences using UniProt and NIH alignment tools reveals that all relevant amino acids interacting with the residues Y453 and N501 are conserved in both ACE2 proteins.; **Supplementary Figure S5.** Binding assays for recombinant SARS-CoV-2 RBD spike mutants. (**A**) Schematic representation of binding assay. (**B**–**D**) Binding assay comparison between mammalian cell lines. Vero E6 TMPRSS2 cells and A549 ACE2 were incubated with (**B**) rSARS-CoV-2 WT, (**C**) rSARS-CoV-2 Y453F or (**D**) rSARS-CoV-2 N501Y at 4 °C to evaluate the interaction between the spike and ACE2 receptor. Samples were evaluated by qPCR for RdRp and compared between cell lines. Quantification relative to 18S rRNA. n  =  3 independent experiments with two replicates each. The graphic shows mean values ± SD. Asterisks above the bars indicate the degree of significance compared to the control condition (* = *p* < 0.05, ** = *p* < 0.01, *** = *p* < 0.001, **** = *p* < 0.0001, *ns* = not significant by one-way ANOVA).; **Supplementary Figure S6.** Competition assay for recombinant SARS-CoV-2 RBD spike mutants. (**A**) Experimental design for competition assay. (**B**,**C**) Controls for competition assay. (**B**) Vero E6 TMPRSS2 and (**C**) A549 ACE2 cells were infected at MOI 0.1 PFU/cell with rSARS-CoV-2 WT, rSARS-CoV-2 N501Y and rSARS-CoV-2 Y453F separately. After 48 hpi, RNA from supernatants were collected and proceded for sequencing by Oxford Nanopore.; **Supplementary Figure S7.** NSP9 binding sites by covalent RNA immunoprecipitation. U76G-dependent changes in covalent NSP9-RNA linkages across the SARS-CoV-2 genome in positive and negative-sense RNA. Representative experiment in Vero E6 TMPRSS2 cells (rSARS-CoV-2 WT vs. rSARS-CoV-2 U76G) at 24 hpi is shown. One out of two independent experiments shown.; **Supplementary Table S1.** List of oligonucleotides used in the present study.; **Supplementary Table S2.** List of mutations during rSARS-CoV-2 sequencing with more 50% presence.
