# Supplementary material for: High-Fidelity and Cost-Effective Engineering of SARS-CoV-2
Source: Viruses. 2025 Dec 11;17(12):1604. doi: 10.3390/v17121604 (PMC12737603; doi:10.3390/v17121604)
Supplement: Supplementary file 1 [file viruses-17-01604-s001.zip › viruses-4001899-supplementary.pdf]

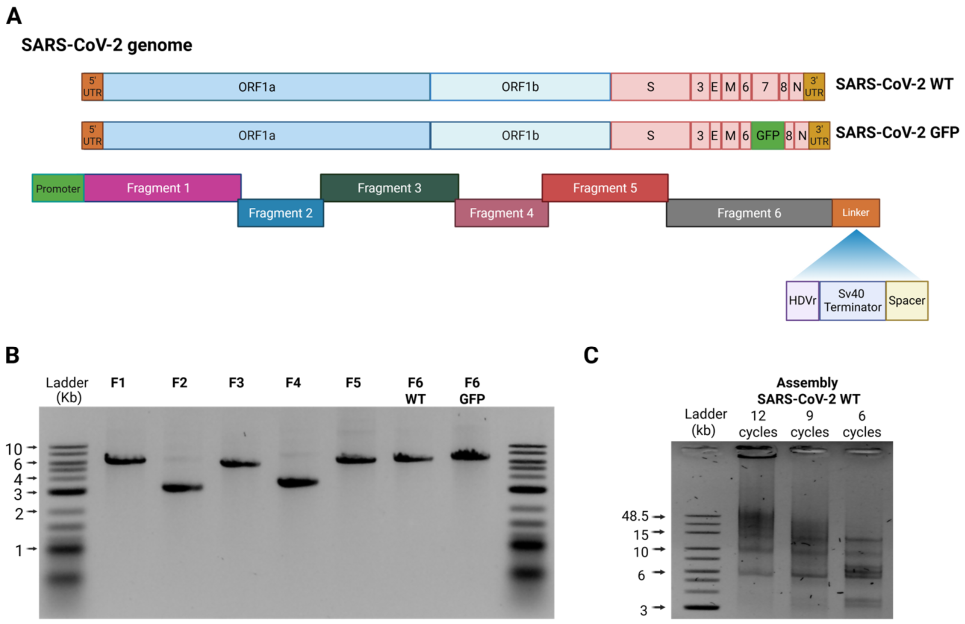


**Supplementary Figure S1.** General overview and design of PCR-based assembly for SARS-CoV-2 DNA. (**A**) Schematic representation of SARS-CoV-2 WT and rSARS-CoV-2 GFP. Some modifications were introduced into the SARS-CoV-2 genome. First, a CMV promoter was added to the upstream region of the 5′ UTR in fragment F1. Next, a linker sequence was introduced after the 3′ UTR in fragments F6 WT and F6 GFP consisting of: 1) Hepatitis delta virus ribozyme: cleavage of the phosphodiester bound for a precise 3′ end of the viral RNA; 2) SV40 Poly a terminator: ending of transcription to avoid the generation of concatemeric RNA and 3) a Spacer sequence: 350 bp to create an intermediate area between SV40 and the CMV transcription promoter during the circu-larization assembly. (**B**) DNA fragments of SARS-CoV-2. PCR products of 7 fragments of SARS-CoV-2, including the fragment for the GFP reporter, using bacterial plasmids as template. (**C**) Optimization of assembly for SARS-CoV-2 DNA. Three different assembly conditions (12, 9 and 6 polymerase extension cycles) were tested using an equimolar mixture of 6 DNA fragments to assemble the 30 kb SARS-CoV-2 genome.

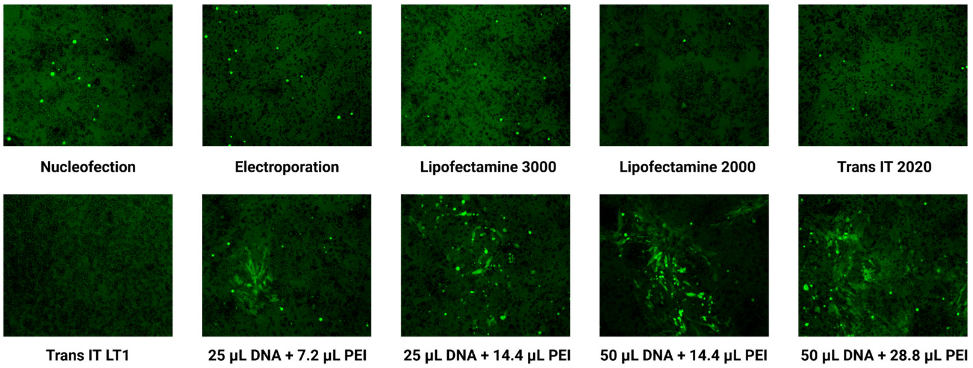


**Supplementary Figure S2.** Gene delivery for rescue of rSARS-CoV-2. SARS-CoV-2 DNA assembly and N plasmid were nucleofected, electroporated or lipofected using different transfection rea-gents

 https://doi.org/10.3390/v17121604

into HEK293T ACE2 cells. After 24 h, cells were trypsinized and seeded over a monolayer Vero E6 TMPRSS2 cells. After 10 days, expression of GFP was evaluated as a sign of rescue of rSARS-CoV-2 GFP.

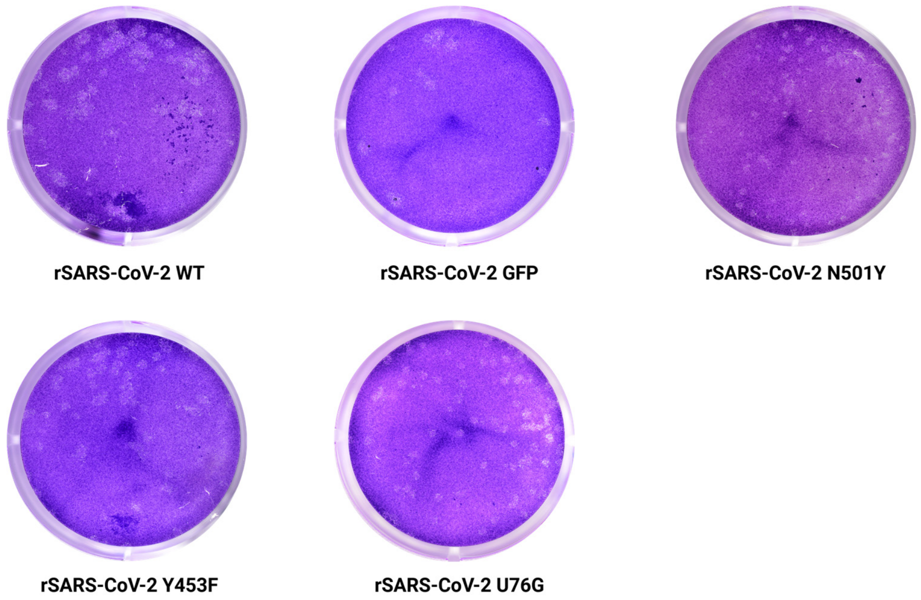


**Supplementary Figure S3.** Plaque morphology of recombinant SARS-CoV-2. Representative plaque morphology of rSARS-CoV-2 WT, rSARS-CoV-2 GFP, rSARS-CoV-2 N501Y, rSARS-CoV-2 Y453F, and rSARS-CoV-2 U76G in Vero E6 TMPRSS2 at 72 hpi.

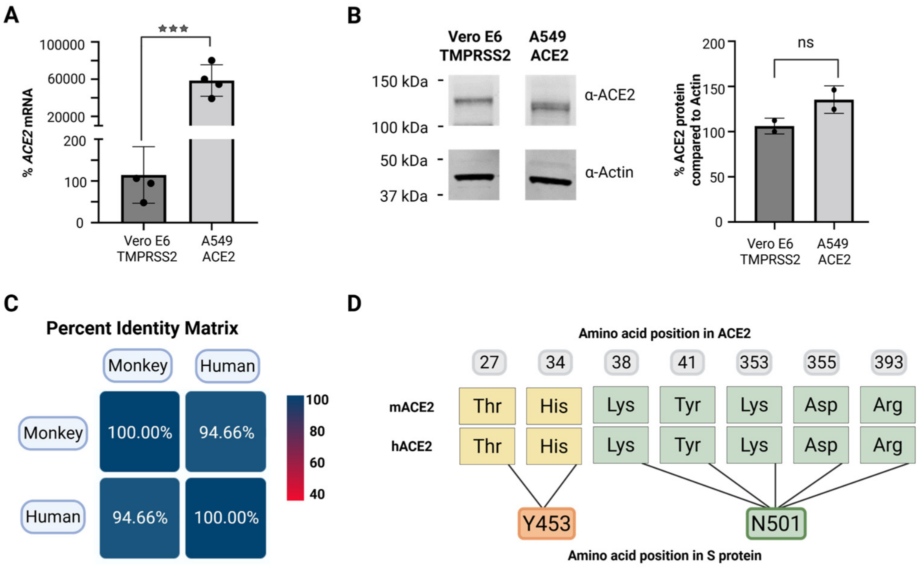


**Supplementary Figure S4.** Characterization of ACE2 in mammalian cells. (**A**) RT-qPCR analysis of ACE2 mRNA levels in Vero E6 TMPRSS2 and A549 ACE2 as indicated. Quantification relative to 18S rRNA and Vero E6 TMPRSS2 cells. n = 2 independent experiments with two replicates each. The graphic shows mean values ± SD. Asterisks indicate the degree of significance compared to Vero E6 TMPRSS2 cells (*=p<0.05, **= p<0.01, ***= p<0.001, ****= p<0.0001 by two-sided unpaired Student's t-test). (**B**) Western blot analysis of ACE2 in Vero E6 TMPRSS2, A549 ACE2 and A549 cells. Actin serves as control. Quantification of ACE2 protein levels in two replicate experiments is shown on

the right. The graphic shows mean values ± SD. Asterisks indicate the degree of significance compared to Vero E6 TMPRSS2 cells (*=p<0.05, **= p<0.01, ***= p<0.001, ****= p<0.0001, *ns* = not significant by two-sided unpaired Student's t-test). (**C**) Comparison of ACE2 protein identity. Protein sequences for hACE2 and mACE2 were compared using UniProt and NIH alignment bioinformatics tools. The percentage inside the box represents the identity between the proteins. (**D**) Comparison of amino acid residues critical for binding with the residues Y453 and N501 in the S protein. Comparison of hACE2 and mACE2 protein sequences using UniProt and NIH alignment tools reveals that all relevant amino acids interacting with the residues Y453 and N501 are conserved in both ACE2 proteins.

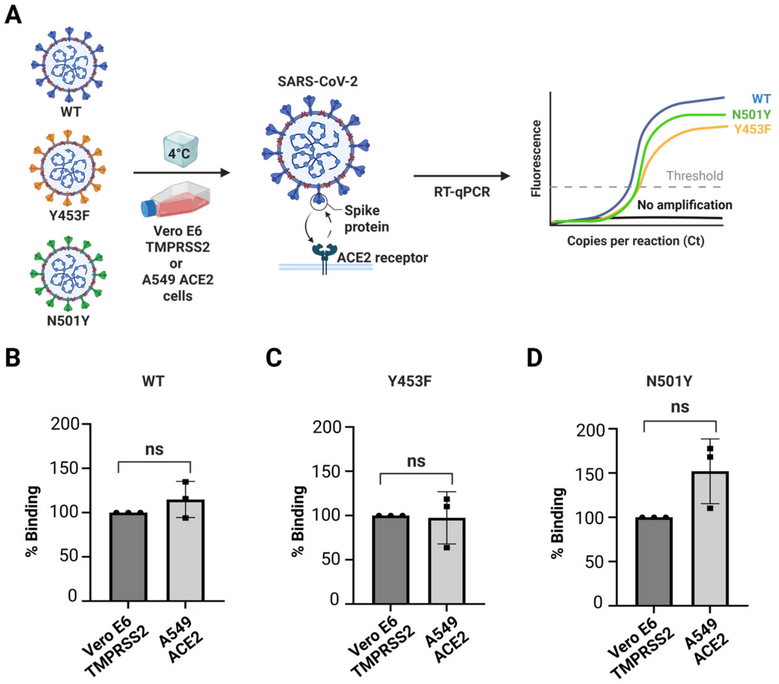


**Supplementary Figure S5.** Binding assays for recombinant SARS-CoV-2 RBD spike mutants. (**A**) Schematic representation of binding assay. (**B**, **C** and **D**) Binding assay comparison between mammalian cell lines. Vero E6 TMPRSS2 cells and A549 ACE2 were incubated with (**B**) rSARS-CoV-2 WT, (**C**) rSARS-CoV-2 Y453F or (**D**) rSARS-CoV-2 N501Y at 4°C to evaluate the interaction between the spike and ACE2 receptor. Samples were evaluated by qPCR for RdRp and compared between cell lines. Quantification relative to 18S rRNA. n = 3 independent experiments with two replicates each. The graphic shows mean values ± SD. Asterisks above the bars indicate the degree of significance compared to the control condition (*=p<0.05, **= p<0.01, ***= p<0.001, ****= p<0.0001, *ns* = not significant by one-way ANOVA).

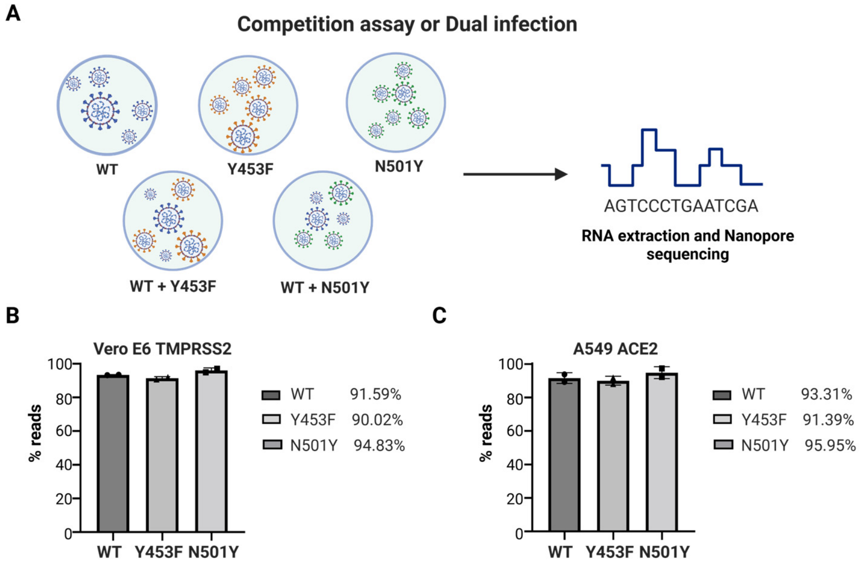


**Supplementary Figure S6.** Competition assay for recombinant SARS-CoV-2 RBD spike mutants. (**A**) Experimental design for competition assay. (**B** and **C**) Controls for competition assay. (**B**) Vero E6 TMPRSS2 and (**C**) A549 ACE2 cells were infected at MOI 0.1 PFU/cell with rSARS-CoV-2 WT, rSARS-CoV-2 N501Y and rSARS-CoV-2 Y453F separately. After 48 hpi, RNA from supernatants were collected and proceded for sequencing by Oxford Nanopore.

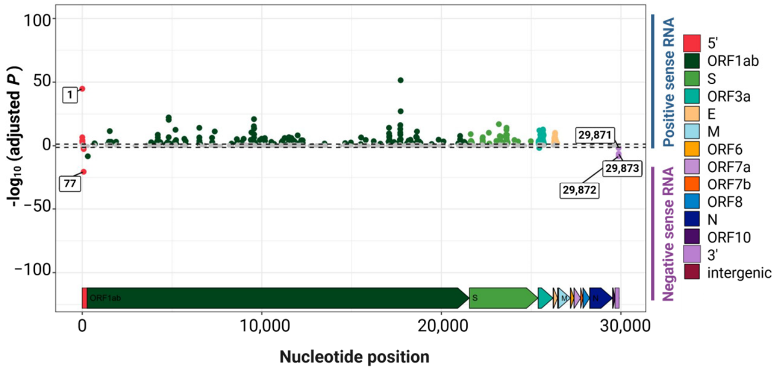


**Supplementary Figure S7.** NSP9 binding sites by covalent RNA immunoprecipitation. U76G-dependent changes in covalent NSP9-RNA linkages across the SARS-CoV-2 genome in positive and negative-sense RNA. Representative experiment in Vero E6 TMPRSS2 cells (rSARS-CoV-2 WT vs. rSARS-CoV-2 U76G) at 24 hpi is shown. One out of two independent experiments shown.

**Supplementary Table S1.** List of oligonucleotides used in the present study.

| Name | Sequence |
|---|---|
| Cloning_F1_SARS2_FW | CTTGGATCCGGTCTCGATTAAAGGTTTATACCTTCCCAG |
| Cloning_F1_SARS2_RV | GGTGGTACCGGTCTCACATCATAACAAAAGGTGACTC |
| Cloning_F2_SARS2_FW | CTTGGATCCGGTCTCTGATGTCAGCACCACCTGCTCAGTA |
| Cloning_F2_SARS2_RV | CTTGGTACCGGTCTCACCAGGCACGACAAAACCCACTTC |
| Cloning_F3_SARS2_FW | CCTTGGATCCAATTGGTCTCCCTGGTTTGCCTGGCACGATATTAC |
| Cloning_F3_SARS2_RV | TTAAGAGCTCAATTGGTCTCCTCTGAAGTGGTATCCAGTTGAAACTACAAATGG |

| | |
|---|---|
| Cloning_F4_SARS2_FW | CATCGGATCCAATTGGTCTCTCAGAGAGCTAGGTGTTGTACATAATCAG-GATG |
| Cloning_F4_SARS2_RV | CAGTGGTACCGCAACTTGTCATAAAGGTCTCTATCAGACATTATGC |
| Cloning_F5_SARS2_FW | GATTTCTAGAGCATACTTTGCATAATGTCTGATAGAGACCTTTATGAC |
| Cloning_F5_SARS2_RV | TATAGGATCCGTGCACAAATGAGGTCTCTAGCAGCAATATCAC |
| Cloning_F6_SARS2_FW | CTACCCCGGGGATATTGCTGCTAGAGACCTCATTTG |
| Cloning_F6_SARS2_RV | TAAGAGCTCAATTGGTCTCTTTTTCCCGGGTTTTTTTTTTTTTTTTTTTT-GTCATTCTC |
| Cloning_CMV_FW | CTTGGATCCGGTCTCTTCCGGCCTGACATTGATTATTGACTAGTTATTAA-TAGTAATCAA |
| Cloning_CMV_RV | GGAGTAGGTCTCTTAATCGGTTCACTAAACGAGCTCTGC |
| SARS2_NP_FW | CTTAGGATCCCTCACTATTTGTTTCGCGCCCAGTTGC |
| SARS2_NP_RV | GATCGGATCCCAGCAAGAAAGCGAGCTCTCTTAGGCCTG |
| Seq_SARS2_1 | GTAAAACGACGGCCAG |
| Seq_SARS2_2 | CAGCAAGAAAGCGAGCTCTCTTAGGCCTGAGTTGAGTCAGCAC |
| Seq_SARS2_3 | GCTTCAACAGCTTCACTAGTAGGT |
| Seq_SARS2_4 | ACAGTGCTTAAAAAGTGTAAAAGTGCC |
| Seq_SARS2_5 | ACAGTATTCTTTGCTATAGTAGTCGGC |
| Seq_SARS2_6 | GAACAAAGACCATTGAGTACTCTGGA |
| Seq_SARS2_7 | TACGACAGATGTCTTGTGCTGC |
| Seq_SARS2_8 | TAACATGTTGTGCCAACCACCA |
| Seq_SARS2_9 | ACTATGGTGATGCTGTTGTTTACCG |
| Seq_SARS2_10 | ACCAGGCAAGTTAAGGTTAGATAGC |
| Seq_SARS2_11 | ACAAATCCAATTCAGTTGTCTTCCTATTC |
| Seq_SARS2_12 | TGTGTACAAAAACTGCCATATTGCA |
| Seq_SARS2_13 | ACTAGCACTCTCCAAGGGTGTT |
| Seq_SARS2_14 | AGGTTCCTGGCAATTAATTGTAAAAGG |
| Seq_SARS2_15 | ATCAGAGGCTGCTCGTGTTGTA |
| Seq_SARS2_16 | TGGTGTATACGTTGTCTTTGGAGC |
| Seq_SARS2_17 | AGGAATTACTTGTGTATGCTGCTGA |
| Seq_SARS2_18 | ACACTATGCGAGCAGAAGGGTA |
| Seq_SARS2_19 | TGTTAAGCGTGTTGACTGGACT |
| Seq_SARS2_20 | TGACCTTCTTTTAAAGACATAACAGCAG |
| Seq_SARS2_21 | CCAGCAACTGTTTGTGGACCTA |
| Seq_SARS2_22 | AGGTGTGAGTAAACTGTTACAAACAAC |
| Seq_SARS2_23 | TCACTACCAAGAGTGTGTTAGAGGT |
| Seq_SARS2_24 | TTCTCCTAAGAAGCTATTAAAATCACATGG |
| Seq_SARS2_25 | GGCTAGATCTGAGGACAAGAGGGCAAAAGT |
| Seq_SARS2_26 | TTCAGCCCCTATTAAACAGCCTGCACGTGT |
| Seq_SARS2_27 | GCAATCCATCTTGTTCAATCATGC |
| Seq_SARS2_28 | GCAAAACCCGTACCCTAGG |
| Seq_SARS2_29 | GACACTAAGAGGGGTGTATACTGC |
| Seq_SARS2_30 | TACAACACGAGCAGCCTCTGAT |
| Seq_SARS2_31 | GCTTGAAGAGAAGTTTAAGGAAGG |
| Seq_SARS2_32 | GGATGAAGAAGAAGGTGATTGTGA |
| Seq_SARS2_33 | GGAAGAAACTAAGTTCCTCACAGA |
| Seq_SARS2_34 | GCTGATGATTTAAACCAGTTAACTGG |
| Seq_SARS2_35 | ATAGCTGAAATCGGGGCCATTT |
| Seq_SARS2_36 | CGCTGATTTTGCAGATGATTCTTC |

| | |
|---|---|
| Seq_SARS2_37 | TCTAAAGTTGCGTAGTGATGTGCT |
| Seq_SARS2_38 | ATGGTGCTAGGAGAGTGTGGAC |
| Seq_SARS2_39 | TGAGGATCTGAAAACTTTGTCAGG |
| Seq_SARS2_40 | CGTTTAGCTAGTCCAATCAGTAGAT |
| Seq_SARS2_41 | TTCGATTGTGTGCGTACTGC |
| Seq_SARS2_42 | ACCTCTAACACACTCTTGGTAGTGA |
| Seq_SARS2_43 | AGCAAAGCAAGAGCAGCATC |
| Seq_SARS2_44 | TGGATCACCGGTGGAATTGCTA |
| Seq_SARS2_45 | GTGCTTTTTAGCCTTTCTGCTATTC |
| Seq_SARS2_46 | ACTGAGGGAGCCTTGAATACAC |
| Seq_SARS2_47 | CCCTCACCTTATGGGTTGGG |
| Seq_SARS2_48 | GTGATGTTGATATGACATGGTCGT |
| Seq_SARS2_49 | CTGACTTCAGTACATCAAACG |
| SARS2_N501Y_RV | CCAACCCACTTATGGTGTTGGTTACCAACC |
| SARS2_N501Y_RV | GGTAACCAACACCATAAGTGGGTTGGAAACC |
| SARS2_Y453F_RV | GGTAATTATAATTACCTGTTTAGATTGTTTAGGAAGTCTAATC |
| SARS2_Y453F_FW | CCTAAACAATCTAAACAGGTAATTATAATTACCACCAACC |
| SARS2_T76G_FW | CTCTAAACGAACGTTAAAATCTGTGTGG |
| SARS2_T76G_RV | CAGATTTTAACGTTCGTTTAGAGAACAGATC |
| PCR1CMV_SARS2_FW | CTTGGTTTCCGGCCTGACATTGATTATTGACTAG |
| PCR1CMV_SARS2_RV | GGTGCTGACATCATAACAAAAGGTGACTCCTGTTG |
| PCR2_SARS2_FW | CCTTTTGTTATGATGTCAGCACCACCTGCTCAG |
| PCR2_SARS2_RV | GGCAAACCAGGCACGACAAAACCCACTTCTC |
| PCR3_SARS2_FW | GTTTTGTCGTGCCTGGTTTGCCTGGCACGAT |
| PCR3_SARS2_RV | CTAGCTCTCTGAAGTGGTATCCAGTTGAAACTACAAATGG |
| PCR4_SARS2_FW | CTGGATACCACTTCAGAGAGCTAGGTGTTGTACAT |
| PCR4_SARS2_RV | GGTCTCTATCAGACATTATGCAAAGTATGCCTACTTTTG |
| PCR5_SARS2_FW | CTTTGCATAATGTCTGATAGAGACCTTTATGACAAG |
| PCR5_SARS2_RV | GCACAAATGAGGTCTCTAGCAGCAATATCACCA |
| PCR6-LNK_SARS2_FW | GCTGCTAGAGACCTCATTTGTGCACAAAAGTTTAAC |
| PCR6-LKCMV_SARS2_RV | CAATGTCAGGCCGGAAACCAAGAGAGGCCGG |
| 18s_qPCR_FW | CGAGGATCCATTGGAGGGC |
| 18s_qPCR_RV | CCGCTCCCAAGATCCAACT |
| NP_qPCR_SARS2_FW | CACATTGGCACCCGCAATC |
| NP_qPCR_SARS2_RV | GAGGAACGAGAAGAGGCTTG |
| RdRp_qPCR_SARS2_FW | GTGARATGGTCATGTGTGGCGG |
| RdRp_SARS2_RV | CARATGTTAAASACACTATTAGCATA |
| SARS2_leader_FW: | CCCAGGTAACAAACCAACCAAC |
| SARS2_ORF1a(gRNA)_RV | CTCGTTGAAACCAGGGACAAG |
| SARS2_M(sgRNA)_RV | GGTTCCATTGTTCAAGGAGCTT |
| SARS2_N(sgRNA)_RV | GTAATGCGGGGTGCATTTCG |
| ACE2hm_Fw | GCATTTAAAATCCATTGGTCTTCTGTCACC |
| ACE2hm_Rv | AGACCATCCACCTCCACTTCTC |
| TMPRSS2_Fw | CCTCTAACTGGTGTGATGGCGT |
| TMPRSS2_Rv | TGCCAGGACTTCCTCTGAGATG |
| SARS2_2569_A1RV | GCTTCAACAGCTTCACTAGTAGGT |
| SARS2_6847_A2RV | ACAGTATTCTTTGCTATAGTAGTCGGC |
| SARS2_11049_A3RV | GAACAAAGACCATTGAGTACTCTGGA |
| SARS2_15225_A4RV | TAACATGTTGTGCCAACCACCA |

| | |
|---|---|
| SARS2_19254_A5RV | ACCAGGCAAGTTAAGGTTAGATAGC |
| SARS2_23823_A6RV | TGTGTACAAAAACTGCCATATTGCA |
| SARS2_28146_A7RV | AGGTTCCTGGCAATTAATTGTAAAAGG |
| SARS2_4429_B1RV | AGTTTCCACACAGACAGGCATT |
| SARS2_8828_B2RV | CACTTCTCTTGTTATGACTGCAGC |
| SARS2_12780_B3RV | CCTACCTCCCTTTGTTGTGTTGT |
| SARS2_17131_B4RV | ACACTATGCGAGCAGAAGGGTA |
| SARS2_21428_B5RV | TGACCTTCTTTTAAAGACATAACAGCAG |
| SARS2_25647_B6RV | AGGTGTGAGTAAACTGTTACAAACAAC |
| SARS2_29837_B7RV | TTCTCCTAAGAAGCTATTAAAATCACATGG |
| SARS2_31_A1FW | ACCAACCAACTTTCGATCTCTTGT |
| SARS2_4295_A2FW | ACAGTGCTTAAAAAGTGTAAAAGTGCC |
| SARS2_8596_A3FW | ACTTGTGTTCCTTTTTGTTGCTGC |
| SARS2_12711_A4FW | TACGACAGATGTCTTGTGCTGC |
| SARS2_16847_A5FW | ACTATGGTGATGCTGTTGTTTACCG |
| SARS2_21358_A6FW | ACAAATCCAATTCAGTTGTCTTCCTATTC |
| SARS2_25602_A7FW | ACTAGCACTCTCCAAGGGTGTT |
| SARS2_1876_B1FW | ATCAGAGGCTGCTCGTGTTGTA |
| SARS2_6287_B2FW | TGGTGTATACGTTGTCTTTGGAGC |
| SARS2_10363_B3FW | TGTTCGCATTCAACCAGGACAG |
| SARS2_14546_B4FW | AGGAATTACTTGTGTATGCTGCTGA |
| SARS2_18897_B5FW | TGTTAAGCGTGTTGACTGGACT |
| SARS2_23123_B6FW | CCAGCAACTGTTTGTGGACCTA |
| SARS2_27447_B7FW | TCACTACCAAGAGTGTGTTAGAGGT |
| SARS2_gfp_27407_B7FW | TTTTCTTGGCACTGATAACACTCG |

**Supplementary Table S2.** List of mutations during rSARS-CoV-2 sequencing with more 50% presence.

| Virus | Passage | Independent Rescue | Position | Reference base | Mutation | Transversion (TV) or Transition (TS) | % Mutation Presence | Amino acid change | Protein altered |
|---|---|---|---|---|---|---|---|---|---|
| WT | 1 | 1 | 29662 | T | C | TS | 92.34 | 3′ UTR | - |
| WT | 1 | 2 | 3426 | C | T | TS | 94.22 | P236L | NSP3 |
| | 1 | 2 | 7299 | C | T | TS | 97.03 | A1527V | NSP3 |
| | 1 | 2 | 9698 | A | G | TS | 98.41 | I382V | NSP4 |
| | 1 | 2 | 28320 | C | T | TS | 95.59 | T17M | N |
| WT | 1 | 3 | 5173 | C | T | TS | 50.85 | Syn. | - |
| | 1 | 3 | 5884 | C | T | TS | 52.10 | Syn. | - |
| WT | 1 | 4 | 3199 | T | C | TS | 55.66 | Syn. | - |
| WT | 2 | 1 | 6258 | C | G | TV | 97.35 | T1180S | NSP3 |
| | 2 | 1 | 6633 | C | G | TV | 96.61 | A1305G | NSP3 |
| | 2 | 1 | 23663 | G | A | TS | 96.40 | A701T | S |
| | 2 | 1 | 28810 | C | T | TS | 96.35 | Syn. | - |
| GFP | 1 | 1 | 25962 | T | C | TS | 70.66 | Syn. | - |
| GFP | 2 | 1 | 5896 | A | T | TV | 86.82 | Syn. | - |
